# Resequencing 545 ginkgo genomes across the world reveals the evolutionary history of the living fossil

**DOI:** 10.1038/s41467-019-12133-5

**Published:** 2019-09-13

**Authors:** Yun-Peng Zhao, Guangyi Fan, Ping-Ping Yin, Shuai Sun, Ning Li, Xiaoning Hong, Gang Hu, He Zhang, Fu-Min Zhang, Jing-Dan Han, Ya-Jun Hao, Qiwu Xu, Xianwei Yang, Wenjie Xia, Wenbin Chen, Han-Yang Lin, Rui Zhang, Jiang Chen, Xiao-Ming Zheng, Simon Ming-Yuen Lee, Joongku Lee, Koichi Uehara, Jian Wang, Huanming Yang, Cheng-Xin Fu, Xin Liu, Xun Xu, Song Ge

**Affiliations:** 1 Laboratory of Systematic & Evolutionary Botany and Biodiversity, College of Life Sciences, Zhejiang University, Hangzhou, 310058 Zhejiang China; 2 BGI-Qingdao, BGI-Shenzhen, Qingdao, 266555 Shandong China; 3 State Key Laboratory of Systematic and Evolutionary Botany, Institute of Botany, Chinese Academy of Sciences, Beijing, 100093 China; 4 University of Chinese Academy of Sciences, Beijing, 100049 China; 5 BGI Education Center, University of Chinese Academy of Sciences, Shenzhen, 518083 Guangdong China; 6 BGI-Shenzhen, Shenzhen, 518083 Guangdong China; 7 State Key Laboratory of Quality Research in Chinese Medicine, Institute of Chinese Medical Sciences, University of Macau, Macau, China; 8 Department of Environment and Forest Resources, Chungnam National University, Daejeon, 34134 Korea; 9 College of Liberal Arts and Sciences, Chiba University, Chiba, 263-8522 Japan; 10 James D. Watson Institute of Genome Sciences, Hangzhou, 310058 Zhejiang China; 11 BGI-Fuyang, BGI-Shenzhen, Fuyang, 236009 Anhui China; 12 China National GeneBank, BGI-Shenzhen, Shenzhen, 518120 Zhejiang China

**Keywords:** Evolutionary genetics, Genome evolution, Genetic variation, Plant evolution

## Abstract

As Charles Darwin anticipated, living fossils provide excellent opportunities to study evolutionary questions related to extinction, competition, and adaptation. Ginkgo (*Ginkgo biloba* L.) is one of the oldest living plants and a fascinating example of how people have saved a species from extinction and assisted its resurgence. By resequencing 545 genomes of ginkgo trees sampled from 51 populations across the world, we identify three refugia in China and detect multiple cycles of population expansion and reduction along with glacial admixture between relict populations in the southwestern and southern refugia. We demonstrate multiple anthropogenic introductions of ginkgo from eastern China into different continents. Further analyses reveal bioclimatic variables that have affected the geographic distribution of ginkgo and the role of natural selection in ginkgo’s adaptation and resilience. These investigations provide insights into the evolutionary history of ginkgo trees and valuable genomic resources for further addressing various questions involving living fossil species.

## Abstract (web summary)

Ginkgo is one of the living fossils from the plant kingdom. Here, authors conduct population genomics analyses to reveal its refugia and demographic history, and provide evidence of multiple anthropogenic introductions of ginkgo from eastern China into different continents.

## Introduction

Despite numerous efforts on investigations of living fossils, mysteries have remained for centuries regarding why living fossils appear essentially unchanged (i.e., in morphological stasis), whether living fossils are evolutionary dead ends and what roles humans have played in the survival and spread of living fossils. The ginkgo tree is an enigmatic living fossil, characterized by morphological stasis with almost no morphological change for at least 200 million years^1,2^. A once-diverse and widespread group of gymnosperms, the ginkgo lineage has survived glaciations as a relic in China, has no current living relatives and has recently been redistributed globally via human-aided introductions^3–5^. As one of the best-known and most distinctive trees worldwide, ginkgo has fascinated humans for centuries by its significance in biology and medicine as well as its power as a source of artistic and religious inspiration^3,5–8^.

Although many investigations on the evolutionary history of this mysterious tree have been undertaken, numerous uncertainties and controversies remain, including the identification of relict populations and potential refugia^4,9–11^, population dynamics in response to Pleistocene climate change^9,12,13^, the roles that humans have played in the dispersal of ginkgo trees in China^6,13–15^ and when and how ginkgo trees were introduced to Japan/Korea, Europe, and North America^4–6^ as well as the potential factors contributing to the persistence and resilience of the species^1,5,16^. Exploration of these questions has been impeded by difficulties in obtaining sufficient samples that represent the entire gene pool of this living fossil and the limited genetic markers available for such an isolated gymnosperm species. Given that the ginkgo draft genome was completed^16^, genome-wide resequencing of ginkgo trees across the world offers an unprecedented opportunity to reveal the evolutionary history of ginkgo, which would provide further insights into the extinction, adaptation, and resilience of living fossils, and eventually facilitate the conservation and management of rare and endangered species in general.

In this study, we resequence 545 ginkgo genomes and uncover the evolutionary history of this living fossil. Taking advantage of nuclear and chloroplast genomic data, we investigated unresolved debates on the evolutionary history of ginkgo^3–5^. Specifically, we identify four ancient genetic components and three glacial refugia of ginkgo populations in China. Our demographic analyses detect multiple cycles of population expansion and reduction along with glacial admixture of relict populations in northern China in response to Pleistocene climate change. We also present a scenario on how ginkgo trees have been dispersed out of refugia and out of China based on multiple lines of evidence. Particularly, we characterize the major bioclimatic variables that have shaped the geographic distribution of ginkgo and addressed the potential roles of natural selection in the survival and resilience of ginkgo populations. These investigations lay an important foundation for further studies of the potential mechanism of ginkgo’s survival and resilience.

## Results

### A largest genome dataset for a non-model species

**Fig. 1:**
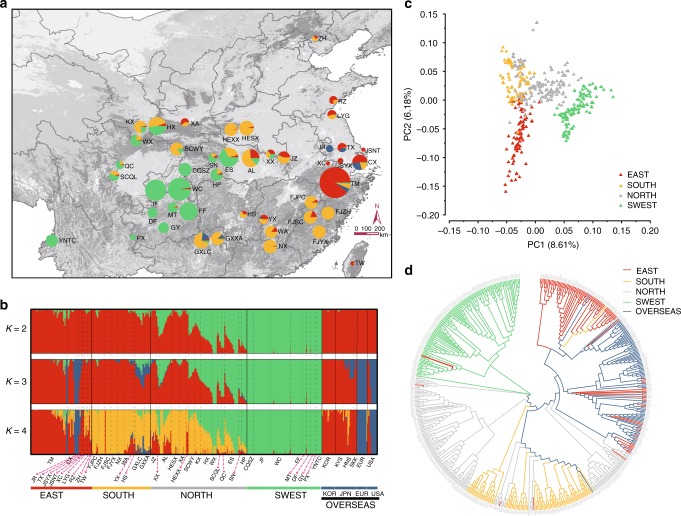
Phylogenetic relationships and population structure of ginkgo populations. **a** Geographic distribution of the sampling locations. The radius of the pies represents population size, and the colors represent ancestral components (according to the substructure at *K* = 4). The map image, derived from ArcGIS Online maps, is the intellectual property of Esri, which is permitted for free use in academic publications (https://doc.arcgis.com/en/arcgis-online/reference/static-maps.htm). Same applies to the other maps in this paper. **b** Model-based population assignment by ADMIXTURE analysis for *K* = 2–5. The *x-*axis shows populations, and the *y*-axis quantifies the proportion of inferred ancestral lineages. **c** Principal component analysis (PCA) of Chinese samples, with the proportion of the variance explained being 8.61% for PC1 and 6.18% for PC2. **d** A neighbor-joining (NJ) phylogenetic tree of all 545 ginkgo samples around the world constructed using whole-genome SNP data based on pairwise identity-by-state (IBS) genetic distances. The source data of Fig. 1a–c are provided in a Source Data file

We have made the most extensive collections of the ginkgo samples around the world to date, including old ginkgo trees from most recorded localities (Supplementary Note 1). In the present study, we sampled 545 ginkgo individuals from 51 populations, covering almost all the natural range and growing locations of ginkgo across the world, which represents the most extensive collection of ginkgo samples to date (Fig. 1a, Supplementary Fig. 1, Supplementary Data 1, and Supplementary Table 1). These samples were sequenced using BGISEQ-500 sequencing platform at an average sequencing depth of ~6.3-fold, generating 44.30 Tb data in total (Supplementary Data 2 and Supplementary Fig. 2). Using the chromosome-level ginkgo reference genome^17^, we obtained 214.33 million raw SNPs and 161.04 million high quality SNPs (Dataset 0) using a strict filtering standard (Supplementary Note 2). Distribution of SNP density along chromosomes, minor allele frequency (MAF), gene location, and inter-SNP distances unraveled the genomic landscape of ginkgo whole-population SNPs (Supplementary Fig. 3). To further assess the performance of the BGISEQ-500 sequencing platform for large genomes, we sequenced 14 individuals randomly selected from Chinese populations using the Illumina sequencing platform. More than 98.6% of SNPs were shared between the two sequencing platforms (Supplementary Table 2), suggesting robust stability of the BGISEQ-500 sequencing platform.

### Four ancient genetic components and three refugia of ginkgo

We used multiple approaches to uncover ancient genetic components of ginkgo and infer the genetic structure and relationships of ginkgo populations (Supplementary Note 4). ADMIXTURE analysis^18^ indicates that the deepest splits (*K* = 2) occurred between the southwestern populations (the SWEST lineage) and the eastern populations (the EAST lineage) plus the southern populations (the SOUTH lineage) (Fig. 1b and Supplementary Fig. 4). It is evident when *K* = 4 that the EAST and SOUTH lineages further diverged and that four ancestral genetic components of ginkgo occur in China, with the northern populations (the NORTH lineage) being admixed with three ancient components (Fig. 1a, b and Supplementary Fig. 4). Consistent with ADMIXTURE results, a principal component analysis (PCA) of Chinese samples reveals three major clusters corresponding to the EAST, SWEST and SOUTH lineages, with the NORTH lineage scattered between them (Fig. 1c and Supplementary Fig. 5). A neighbor-joining (NJ) tree further supports such a pattern (Fig. 1d, Supplementary Figs. 6 and 7), i.e., the SWEST, EAST and SOUTH lineages form three major clades while the NORTH lineage consists of multiple subclades divided into three major clades. These results indicate that the Chinese ginkgo populations harbor four ancient genetic components and consist of four major lineages in southwestern, southern, eastern and northern China. In addition to the well-known refugium in southwestern China^9–11^, we identified a second refugium in eastern China^9–13^ and a third potential refugium in southern China where three ancient genetic components were uncovered (Fig. 1a and Supplementary Fig. 4). It is not unexpected to identify three refugia for ginkgo because these areas are biodiversity hotspots and were the Pleistocene refugia for many seed plants^19–21^, including other living fossils such as *Cathaya argyrophylla*^22^, *Davidia involucrata*^23^, and *Metasequoia glyptostroboides*^24^.

Phylogenetic analysis based on a plastome dataset, representing the nonrecombinant and maternally inherited genome, showed that 446 Chinese ginkgo trees formed three major clades with significant genetic divergence between them (Supplementary Fig. 8). The first clade consisted exclusively of the individuals from the EAST lineage, and the second comprised mostly the individuals from the SWEST lineage. The third and largest clade included the individuals from all four lineages (i.e., the EAST, SOUTH, NORTH, and SWEST lineages) (Supplementary Fig. 8), which was consistent with the results of previous studies based on chloroplast markers^12–14^. Pairwise *F*_ST_ values calculated based on plastome dataset also revealed deep divergence among the lineages with a large number of identical sequences both within and between lineages (Supplementary Table 3), suggesting seed-mediated and long-distance dispersals of ginkgo trees in China possibly due to human activities. Further network construction showed that 13 haplotypes could be divided into three major and distinct groups differing by at least five substitutions, with four major haplotypes in the largest group shared by all lineages (Supplementary Fig. 9), a pattern similar to that revealed by phylogenetic analysis.

A comparably high level of genetic diversity (1.84–2.14 × 10^−3^ for Watterson’s estimator (*θ*_w_) and 2.19–2.41 × 10^−3^ for the average pairwise diversity within populations (*π*) was observed for all four lineages of Chinese ginkgo populations (i.e., EAST, SOUTH, NORTH, and SWEST) (Table 1). Diversity estimates based on the whole chloroplast genomes yielded largely congruent results, i.e., the highest diversity for SWEST and the lowest for SOUTH (Supplementary Table 4). These results are in agreement with those of many previous reports indicating high level of genetic diversity in ginkgo at both the species and local scales^9,10,13^. This result provides additional evidence that no correlation exists between genetic diversity and morphological variation in living fossils^8^. Notably, high genetic diversity was observed in the NORTH lineage (*θ*_w_ = 2.11 × 10^−3^ and *π* = 2.57 × 10^−3^), which reflects the characteristics of admixed origin of ginkgo populations in northern and central China and supports the argument of glacial admixture of relict populations in many species in eastern Asia^25^.

**Table 1:** Sample size and genetic diversity of five lineages of ginkgo trees^a^

Lineage	Region	No. populations/no. trees	No. SNPs	π(SD) (10^−3^)	*θ*w(SD) (10^−3^)
EAST	Eastern China	11/96	126,719,717	1.84 (0.84)	2.41 (0.93)
SOUTH	Southern China	10/92	124,187,675	2.07 0.98)	2.38 (0.92)
NORTH	Northern China	15/152	145,809,400	2.11 (0.95)	2.57 (0.93)
SWEST	Southwestern China	9/118	119,088,666	2.14 (1.00)	2.19 (0.88)
OVERSEAS	Out of China	6/87	116,979,767	2.05 (0.96)	2.26 (0.90)
Total	Global	51/545	160,887,036	2.11 (0.95)	2.36 (0.82)

*π* average number of pairwise nucleotide differences per site, *θ*_W_ Watterson’s estimator of *θ* per base pair

^a^All samples were grouped based on geographical distributions

### Multiple cycles of expansion and reduction of ginkgo populations

**Fig. 2:**
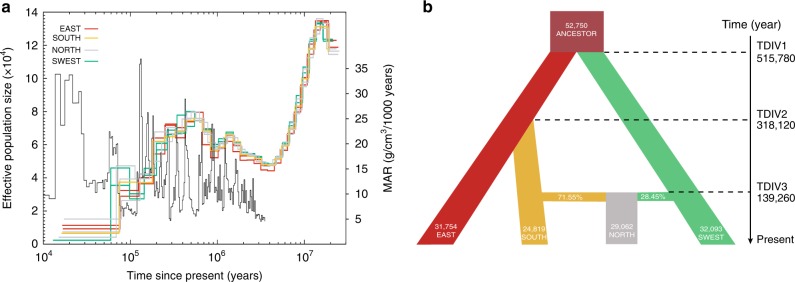
Demographic history of ginkgo populations. **a** Demographic history of four ginkgo lineages including the populations in eastern China (EAST), southern China (SOUTH), northern China (NORTH), and southwestern China (SWEST) inferred by PSMC. The mass accumulation rate (MAR) of Chinese loess is shown in black line. **b** Schematic of demographic scenarios modeled using *fastsimcoal2*, with the ancestral population shown in brown. Column width represents the relative effective population size, with the NORTH lineage inferred as a mixture of 71.55% of the SOUTH lineage and 28.45% of the SWEST lineage. The numbers on the vertical axis indicate the estimated time of population divergence

An important question about living fossils is whether their extinction is underway^8^. To reconstruct the demographic history of ginkgo, we applied the pairwise sequentially Markovian coalescent (PSMC)^26^ to assess population size changes and obtained a well-defined demographic history from 20 million to 60,000 years ago (Fig. 2a and Supplementary Fig. 10). We found multiple substantial demographic fluctuations, i.e., three peaks in population size at ~15 million years ago (mya), ~1.05 mya, and ~0.5 mya, as well as three significant population bottlenecks at ~4 mya, 0.1 mya, and 0.07 mya. The result indicated a few of cycles of population expansions and reduction in ginkgo populations during the Pleistocene glaciations. Notably, the demographic fluctuations since 2 mya showed opposite changes in the amount of atmospheric dust, as inferred by the mass accumulation rate (MAR) of Chinese loess^27^, which indicates that population size reductions were correlated with a cold climate (high MAR).

Because of the insufficient power of PSMC in inferring more recent demographic history due to the limited number of recombination events in a single genome^28^, we simulated the demographic fluctuations since 0.4 mya (Supplementary Tables 5 and 6) based on SNPs of the ginkgo populations using *fastsimcoal2*^29^. The model with the best scored supports the earliest divergence between the SWEST and EAST + SOUTH lineages being 515,780 years ago followed by the split-off of the SOUTH lineage 318,120 years ago (Fig. 2b and Supplementary Table 5), which are in well agreement with an estimate based on whole-plastid-genome sequence data^12^. It is evident that the NORTH lineage originated 139,260 years ago as a result of admixture between the SWEST (28.45%) and SOUTH (71.55%) lineages (Fig. 2b), probably resulting from repeated range expansions and contraction during the warming and cooling phases since 0.5 mya^12^. A moderate reduction in effective population sizes (*N*_e_) was observed for the four descendant lineages (*N*_e_ = 24,819 ~ 32,093 relative to their most recent common ancestor (*N*_e_ = 50,514) (Fig. 2b), consistent with the PSMC inference (Fig. 2a). No significant recent gene flow was detected among the three ancient lineages (i.e., the EAST, SOUTH, and SWEST lineages).

The plastome dataset also indicated deep divergence among the three ancient lineages with possible seed-mediated dispersal, given that a large number of identical chloroplast sequences were found among lineages (Supplementary Figs. 8 and 9, Supplementary Table 3). The fact that individuals from the NORTH lineage clustered with all three other lineages supported the admixed nature of the NORTH lineage (Supplementary Fig. 8), as indicated by the nuclear genome dataset. Together, we found that ginkgo populations underwent several cycles of expansion and contraction during the Pleistocene glaciations and revealed a moderate reduction in ginkgo populations in refugia associated with admixture of relict populations in northern and central China.

### Human-aided dispersal of ginkgo out of China

**Fig. 3:**
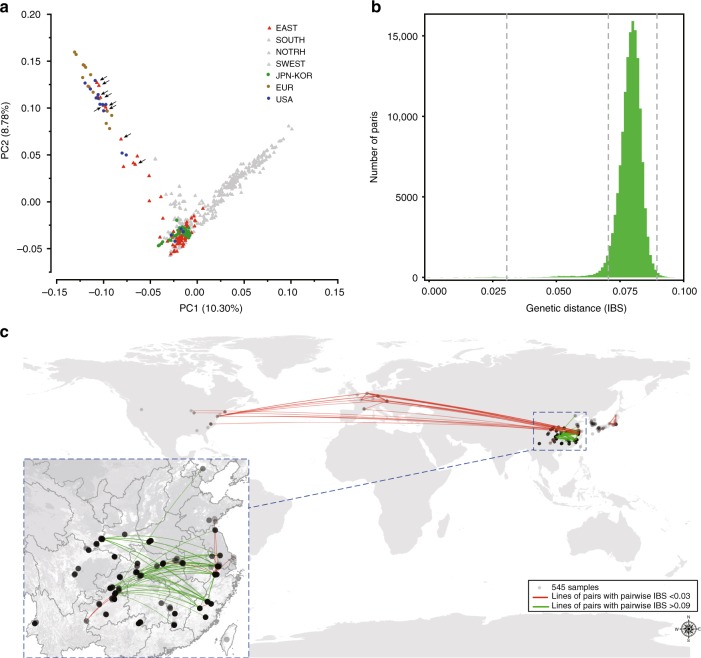
Genetic and geographic distances between all samples. **a** Principal component analysis of global samples. Triangles indicated by black arrows are the samples containing more than 50% of a rare genetic component (blue in Fig. 1b). This component is widespread in the European and American samples. **b** Distribution of pairwise identity-by-state (IBS) genetic distances, with three cutoffs indicted by vertical dashed lines. The map image was derived from ArcGIS. **c** Geographic locations of 545 global samples, showing the pairs with the shortest genetic distance (IBS < 0.03, connected by red lines) and pairs with the longest genetic distance (IBS > 0.09, connected by green lines). All the pairs without a line connection have the IBS values between 0.03 and 0.09. Source data are provided in a Source Data file

Unlike many other living fossils that are confined to an isolated area, ginkgo has been thought to be extinct but has actually flourished after being introduced by humans to different areas around the world^5,6,8^. It remains speculative that ginkgo trees underwent human-aided spread out of the refugia in China and were further introduced to various areas in East Asia, Europe and the Americas^4–6,14^. Our ADMIXTURE result indicates that non-Chinese populations (the OVERSEAS samples) comprise three ancient genetic components that are only found in the EAST and SOUTH lineages (Fig. 3a, Supplementary Fig. 4), excluding the possibility that the SWEST lineage was the donor of ginkgo trees outside of China. PCA and NJ analyses produced congruent results showing that the OVERSEAS samples formed a cluster mainly with EAST that consisted of two subgroups (Fig. 3a and Supplementary Fig. 11). It is noted from Fig. 3a, that the Japanese and Korean samples form a dense cluster with the main group of the EAST samples, while those from Europe are mainly clustered with other EAST samples that have a rare genetic component (Fig. 1b). The American samples form three minor clusters that overlap with those from Japan/Korea, EAST and Europe. These observations support the speculation that old ginkgo trees in the USA might have originated multiple times from eastern China^4,5^ but are inconsistent with the argument that ginkgo in Europe was introduced from Japan^6^.

To better understand the dispersal history of ginkgo trees, we calculated the pairwise identity by state (IBS) genetic distance between individuals (Fig. 3b). We found that the genetic distance between individuals did not follow a strict isolation-by-distance model, in which the genetic distance between individuals correlates with their geographic distance. One extreme case comprising individuals with the smallest IBS values (IBS < 0.03) (Fig. 3b) consists of groups of nearly identical individuals, reflective of recent dispersal or introduction. As shown in Fig. 3c, the pairs with an IBS < 0.03 (red lines) occur mainly among EAST, Europe and the USA, supporting the recent introduction of ginkgo from eastern China to different continents^4,5^. Additional pairs of individuals with an IBS < 0.03 were found in China, Japan, and Europe (Fig. 3c). The pairs with slightly larger IBS values (0.03 ≤ IBS < 0.07, pairs without connected lines) were found mainly between China and Japan/Korea (Fig. 3c), suggesting earlier introductions of Chinese ginkgo into Japan/Korea than into Europe and the USA.

Interestingly, the pairs with the largest IBS values (IBS > 0.09, green lines) only occurred in China, mainly between the EAST and SWEST lineages (Fig. 3c), a reflection of the highest genetic differentiation between the EAST and SWEST lineages Table 1). However, several pairs with almost identical samples (IBS < 0.03, red lines) are also observed in China, linking samples that are geographically distant (Fig. 3c). This result is consistent with the results of analyses based on the plastome dataset, suggesting seed-mediated dispersal (Supplementary Table 3) and supports previous arguments that recent human activity helped in the effective dispersal of ginkgo trees in China^14,15^.

### Environmental adaptation and natural selection

**Fig. 4:**
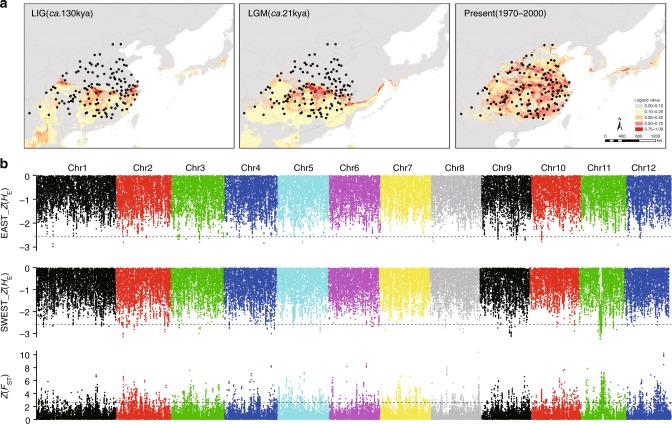
Putative selected regions identified in the EAST and SWEST groups. **a** Predicted distributions of ginkgo trees at different historical periods based on species distribution modeling (SDM). Area color indicates probability (0–1) of suitable habitat for ginkgo. LIG, last interglacial; GM, last glacial maximum. The map image was derived from ArcGIS. **b** Distribution of the *Z*-score-transformed expected heterozygosity (*H*_E_) and *F*_ST_ for EAST-g and SWEST-g in 100 kb nonoverlapping sliding windows along chromosomes. Horizontal dotted lines represent the cutoff fulfilling the requirement for the selected regions. The source data of Fig. 4b are provided in a Source Data file

To reveal the potential impact of climate fluctuations on the distribution of the species, we simulated distribution patterns of ginkgo at present (1970–2000), during the last glacial maximum (LGM: c. 21 thousand years before present, kyr BP), and the last interglacial (LIG: c. 115–130 kyr BP) with all 19 bioclimatic variables using species distribution modeling (SDM)^30^ (Supplementary Note 6). It is evident that the overall distribution pattern of the species predicted for the present is largely consistent with its actual distribution and that suitable habitats for ginkgo contracted during the LGM and then expanded northward after the LGM, with the suitable habitats being much larger at present than during the LGM (Fig. 4a). We then estimated the relative importance of the climatic variables on the species distribution and found that seven bioclimatic variables, i.e., mean temperature of the coldest quarter (BIO11), temperature seasonality (BIO4), mean temperature of the wettest quarter (BIO8), precipitation of the driest month (BIO14), precipitation seasonality (BIO15), precipitation of the warmest quarter (BIO18) and mean diurnal range (BIO2), strongly affected the distribution of ginkgo trees (Supplementary Table 7). Notably, the variables determining the ginkgo distribution differ between eastern and southwestern China (Supplementary Table 7), implying the differentiation of habitat preferences between ginkgo populations in the two refugia.

To examine the potential mechanism of adaptation to environmental changes during ginkgo’s evolution, we investigated genomic signals of adaptation at the local scale. We selected one group of individuals (58 trees) each from the EAST and SWEST lineages, herein named EAST-g and SWEST-g, respectively (Fig. 1a, Supplementary Table 8) to represent the ginkgo lineages in two refugia and searched the genome for potential regions with signatures of selection using parameter-based statistics and a likelihood-based program SweeD^31^ (Supplementary Note 7). In total, we identified 7 windows in EAST-g and 46 windows in SWEST-g, which showed signature of selection (Fig. 4b and Supplementary Data 3) and most of these regions could also be identified under different sliding widows (Supplementary Data 4 and 5). To avoid the potential impact of low genetic variation at the linked loci on the detection of selective signals, we implemented composite likelihood ratio (CLR) statistics to identify regions that are significant deviations from neutral the site frequency spectrum (SFS) (Supplementary Note 7). The results showed that a total of 910 and 949 putative regions that showed signatures of selection and contained 643 and 504 candidate genes, in EAST-g and SWEST-g, respectively (Fig. 4b, Supplementary Data 6 and 7). We further performed gene ontology (GO) enrichment analysis of the genes in these putative regions and obtained 14 and 17 significantly enriched terms (Fisher’s exact test, *P* < 0.05) in EAST-g and SWEST-g, respectively, including many terms involving responses to various abiotic and biotic stress (Supplementary Table 9). Sequence homology analysis of 25 genes identified by both approaches revealed some important genes involved in insect and fungal defenses and responses to abiotic stress such as dehydration, low temperature and high salt (Supplementary Note 7 and Supplementary Table 10), consistent with previous findings that ginkgo possessed unusually high resistance or tolerance to both abiotic and biotic stress, particularly herbivores and pathogens^1,6,16^. Further studies on the function of these genes and their roles in ginkgo adaptation are highly encouraged.

## Discussion

This study uncovered the evolutionary history of a species with a very large genome (10.6 Gb^16^) based on resequencing of hundreds of samples covering all known localities of the species. Here we used an updated genome assembly using HiC technolog^17^ to conduct substantial investigations on population genomics and evolutionary history of ginkgo. Such a chromosome-level reference provides the distribution information of SNPs^17^ and thus represents a valuable resource for addressing numerous questions involving biologic diversity, population genetics, and evolutionary history. To evaluate the applicability of SNPs called based on draft genome where no position information is available, we used the SNPs called based on the draft genome^16^ to perform various analyses. As demonstrated by ADMIXTURE analyses (Supplementary Fig. 4c), PCA and NJ trees of both Chinese (Supplementary Figs. 5c, d and 6b) and global (Supplementary Figs. 11c, d and  7b) samples as well as the distribution of the IBS genetic distances (Supplementary Fig. 12), the two datasets (i.e., the SNPs called based on the draft and updated genomes) generated almost identical results, implying that the SNPs called based on a draft genome (or virtual pseudo-chromosomes by randomly linked scaffolds) could provide sufficient information for addressing various evolutionary questions as long as the SNPs were called under strict filtering standards.

This study revealed three glacial refugia of ginkgo populations and demonstrated the glacial admixture of relict populations of ginkgo in northern China. In addition to the two refugia in eastern and southwestern China reported in the previous studies^10,11,14^, we identified a third refuge in southern China in which all four ancient genetic components were found (Fig. 1 and Supplementary Fig. 1). This area also inhabited an array of other relict tree species such as *Cathaya*, *Metasequoia*, *Davidia*, *Cercidiphyllum*, *Euptelea*, *Glyptostrobus*, and *Tetracentron* survived the Pleistocene glaciations^11,19,20,24,32,33^. The finding of the admixed northern populations (the NORTH lineage) of ginkgo (Figs. 1 and 2) supported the hypothesis that glacial admixture of relict populations occurred in many species in China^25^. Importantly, we detected highly dynamic of population sizes, and particularly significant population expansions and reductions during the Pleistocene glaciations in all refugia (Figs. 2 and 4a), consistent with previous studies on ginkgo^9,12^ and other seed plants^21,32^.

Recent investigations based on SSR markers and chloroplast sequences^12,13^ suggested that both random genetic drift and introgressions between lineages during the cycles of glaciations might have contributed to the current genetic structure of ginkgo populations. The present study supported the deep divergence among the three refugia by both nuclear and chloroplast datasets (Supplementary Figs. 8 and 9). In particular, the high proportion of identical sequences between lineages in maternally inherited chloroplast genome (Supplementary Table 3) implies the presence of introgression or gene flow via seed dispersal and migration, possibly by human activities^5,9,12^.

Ginkgo trees have long been used in diverse ways such as medicine, food, and ornamentation as well as culture and religion and thus have been subjected to human impacts for centuries^3,5,6,11^. However, the roles that humans have played in the survival and spread of ginkgo populations are largely speculative^3,5,11^. The oldest known ginkgo trees in China were estimated to be approximately 1000–3000 years old, with many of them being adjacent to human settlements^5,15^; thus, human-mediated introduction might have occurred at the regional scale in China^3,5,13,14^. The findings of recent long-distance dispersals in both China and Japan, as evidenced by the pairwise identity-by-state (IBS) genetic distances of nuclear genomes (Fig. 3c) and the identical chloroplast sequences (Supplementary Table 3), provide empirical evidence supporting the importance of human-mediated introduction in ginkgo dispersal. There is no doubt that the everlasting interaction between ginkgo and people because of traditional culture and belief helps protect natural ginkgo forests and ultimately ensures ginkgo’s survival and dispersal^5,6,11^.

How ginkgo was introduced into various areas across the continents is another longstanding controversy^4,6^, although it is well acknowledged that ginkgo trees have been increasingly planted across Europe and America since the 18th century^3,5^. Our analyses based on multiple approaches (Figs. 1 and 3) clearly showed multiple introductions of Chinese ginkgo trees into North America and Europe but refuted the possibility that European ginkgo originated from either Japan or Korea^6^. A mystery that has intrigued scientists is why living fossils look unchanged (morphological stasis) with various hypotheses such as ecological conservatism^8,34,35^. Our SDM analysis indicated the habitat persistence in the potential refugia of ginkgo trees (Fig. 4a), in agreement with the fossil evidence^11^. A high level of nucleotide diversity maintained in ginkgo (Table 1) supports the hypothesis of evolutionary capacitance^8,34^, i.e., a species might continually accumulate genetic changes, that are not necessarily accompanied by a similarly steady increase in phenotypic variation. It is likely that the morphological stasis of living fossils is an effective adaptation strategy in response to environmental change, although the underlying mechanisms are unclear^8,34^. The reasons for and potential factors contributing to ginkgo’s resilience have been speculated about in many previous studies, which showed that ginkgo trees maintained outstanding resistance or tolerance to both herbivores and pathogens, partly accounting for the longevity and high vegetative propagation ability of individual trees^1,6,16^. Consistently, our analyses of adaptation revealed numerous pathways and specific genes that are involved in responses to abiotic and biotic stresses (Supplementary Note 7), which deserves further in-depth investigations.

Whether living fossils and relict species are undergoing extinction is an intriguing question. As an example, the giant panda has long been regarded as an evolutionary dead-end but proved to be a successful species highly adapted to environmental changes^36^. Wei et al.^37^ argued that extensive speculation about the giant panda arose from an unsystematic and unsophisticated understanding of its biology. The present study provides multiple lines of evidence explicitly refuting the notion that ginkgo is an evolutionary dead end in terms of adaptation to abiotic and biotic stress (Supplementary Note 7) as well as the high level of genetic diversity (Table 1), repeated resurgence of population size in response to glaciations (Fig. 2), and a wide distribution of potential habitats (Fig. 4a). The dawn redwood tree (*Metasequoia glyptostroboides*) is another famous living fossil that was believed to have been extinct for several million years and re-discovered in the 1940s in several isolated areas in central and southern China^38^. Similar to ginkgo, dawn redwood has successfully expanded its distribution to approximately 50 countries via decades of human efforts, even over a much wider range than the fossils indicated^24^. Living gymnosperms that were considered as ancient and living fossils are convincing examples of successful plant lineages, in which many extant species occupy diverse habitats and survive dramatic environmental changes by adaptive shifts^39,40^. For example, in a study on the cycad lineage, Nagalingum et al.^41^ found that cycads underwent global rediversification beginning in the late Miocene, indicating that the species diversity of today’s cycads arose from a relatively recent radiation, less than 12 mya. Ginkgo’s resilience, along with other similar cases such as that in dawn redwood^24^ and cycads^40,41^ as well as some other gymnosperms^39^ supports the argument that intuitively frangible living fossils may possess the ability to survive successfully and even flourish if they are reintroduced to suitable habitats^8^, particularly when aided by humans. Together, the results of our study provide not only a comprehensive evolutionary framework for ginkgo research and conservation but also insights into the evolutionary history and conservation of other living fossil species.

## Methods

### Sample collection and resequencing

A total of 545 individual ginkgo trees were carefully selected to represent most of the known localities of large ginkgo trees around the world, including 51 populations from nine countries (Supplementary Fig. 1 and Supplementary Note 1). Genomic DNA extraction, library construction, and amplification followed standard protocols (Supplementary Note 1). All samples were sequenced using BGISEQ-500 with a pair-end read length of 50 bp or 100 bp. We filtered raw data using SOAPnuke (ver. 1.5.4)^42^ and obtained clean sequencing reads with an average depth of up to 6.1-fold, ranging from 4- to 10-fold for each sample (Supplementary Data 2, Supplementary Figs. 2, 13).

### SNP calling, quality control, and validation

We used the DRAGEN (https://www.illumina.com/products/by-type/informatics-products/dragen-bio-it-platform.html) toolkit to conduct read mapping and SNP calling, with the clean reads of FASTQ files used for alignment, sorting, duplicate removal, and variant calling. About 99% of the samples were aligned to the reference genome with a mapping ratio of more than 85% (Supplementary Data 2 and Supplementary Fig. 14). The hidden Markov model and Smith-Waterman alignment in GATK 4.0 Haplotype Variant Caller^43^ were used for the SNP calling. The raw SNP dataset was filtered using variant quality score recalibration (VQSR), with the majority of the SNPs showing high quality scores and low missing rates (Supplementary Fig. 15). Five datasets were compiled to satisfy the requirements of various analyses (Supplementary Note 2). To assess potential biases resulting from the use of different sequencing platforms, we sequenced 14 randomly selected samples using the Illumina platform. By comparing the two SNP datasets generated by the Illumina HiSeq2000 sequencing platform and BGISEQ-500, we found that more than 98.6% of SNPs were shared by the two platforms with different filtering standards, suggesting high consistency between the two sequencing platforms (Supplementary Table 2).

### Chloroplast genome assembly and SNP calling

Being maternally inherited in ginkgo^14^, the chloroplast genome provides additional information on population genetics and phylogeography^44^. Thus, we obtained SNP data from chloroplast genomes, representing the maternally inherited markers, to perform analyses of phylogenetic relationships and population genetic structure of ginkgo trees. We filtered raw resequencing data to obtain reads by mapping to the chloroplast sequence AB684440.1 of ginkgo^45^ using BWA and SAMtools. A total of 481 chloroplast genomes were completely assembled using the GetOrganelle pipeline^46^, of which 446 plastomes of Chinese ginkgo trees were used in various analyses. SNP calling and quality control for the chloroplast genomes were performed following processes similar to those mentioned above (Supplementary Note 3).

### Analysis of population genetics and phylogeny

We performed routine analyses of population genetic structure and phylogenetic relationships of ginkgo with both the resequencing and plastome datasets (Supplementary Notes 4 and 5). The population structure and admixture of our global samples were inferred using ADMIXTURE (ver. 1.3.0)^18^ with five replicates and 10-fold cross-validation (CV) from *K* = 2 to 10. PCA was conducted to study the relatedness and clustering of populations or samples. The top 10 PCs of the variance-standardized relationship matrix were extracted using PLINK (ver. 1.90)^47^ with the–pca parameter. To quantify the relatedness between individuals, the pairwise identity-by-state (IBS) genetic distance matrix of all 545 samples was calculated using PLINK^48^ (ver. 1.90) with the parameter–distance 1-ibs. We constructed a neighbor-joining (NJ) phylogenetic tree using MEGA (ver. 4.0)^49^ based on the distance matrix. A haplotype network was constructed for the plastome dataset using PopART^50^ based on codon-based alignments from chloroplast genomes.

### Demographic history inference

PSMC was used to infer historical dynamics of effective population size and the divergence timing of ginkgo lineages^51^ based on 8 samples from four lineages with a high sequencing depth (~30-fold) (Supplementary Table 11). The whole-genome diploid consensus sequences for each sample were generated by SAMtools and BCFtools with the parameter C50. Sites with sequencing depths < 10 and > 100 (vcfutils.pl vcf2fq -d 10 -D 100) were removed to reduce the probability of false positives. The PSMC parameter (psmc -N25 -t15 -r5 -p “4 + 25*2+4+6”) was used to infer the historical effective population size. The estimated generation time and mutation rate were set to 20 and 0.67e−9, respectively (Supplementary Fig. 16). To infer demographic history using *fastsimcoal2*^52^, two-dimensional joint SFS (2D-SFS) was constructed from posterior probabilities of sample allele frequencies using easySFS.py (“https://github.com/isaacovercast/easySFS). Each model was run 100 times with 100,000 simulations for the calculation of the composite likelihood, and 10–40 expectation-conditional maximization (ECM) cycles (Supplementary Fig. 17). Model comparison was based on the maximum likelihood value across 50 independent runs using the Akaike information criterion and Akaike’s weight of evidence^52^. The model with the maximum Akaike weight value was chosen as the optimal model. Finally, we calculated confidence intervals of parameter estimates from 100 para-metric bootstrap replicates by simulating SFS from the maximum composite likelihood estimates and re-estimating parameters each time^53,54^. Based on the pairwise identity by state (IBS) genetic distance calculated by PLINK (ver. 1.9), we used a histogram to display the IBS distribution. Pairs with short genetic distances (IBS < 0.03 or IBS < 0.07) may reflect recent dispersal or introduction.

### Analysis of the species distribution and local adaptation

We performed species distribution modeling (SDM) using the software MAXENT (v3.3.1)^30,55,56^ to explore the impacts of climate on the species distribution and to detect major climatic variables that contribute to the species distribution (Supplementary Note 6). We detected genomic regions under selection in two groups of individuals (EAST-g and SWEST-g), representing the EAST and SWEST lineages, respectively to explore the genomic basis of local adaption using two approaches (Supplementary Note 7, Supplementary Figs. 18 and 19), including one based on population genetics summary statistics (*H*_E_ and *F*_ST_)^57^ and the other in the likelihood-based program SweeD (version 3.1)^58,59^. We performed gene ontology (GO) enrichment analysis for the candidate genes in EAST-g and SWEST-g using a perl script^60^.

### Reporting summary

Further information on research design is available in the Nature Research Reporting Summary linked to this article.

## Supplementary information

Supplementary Information

Peer Review

Reporting Summary

Description of Additional Supplementary Files

Supplementary Data 1

Supplementary Data 2

Supplementary Data 3

Supplementary Data 4

Supplementary Data 5

Supplementary Data 6

Supplementary Data 7

Source Data file

## Data Availability

Data supporting the findings of this study are available within the paper and its Supplementary Information files. The resequencing reads of gingko in this study have been deposited in NCBI Sequence Read Archive (SRA) under BioProject accession PRJNA478810. The datasets reported in this study are also available in the CNGB Nucleotide Sequence Archive under accession number CNP0000136. The source data underlying Figs. 1a–c, 3a–c, 4b and Supplementary Figs. 2, 3b, 5a, b, 6a, 11a, b, 14, 15, and 18 are provided as a Source Data file.
