# Supplementary material for: Resequencing 545 ginkgo genomes across the world reveals the evolutionary history of the living fossil
Source: Nat Commun. 2019 Sep 13;10:4201. doi: 10.1038/s41467-019-12133-5 (PMC6744486; doi:10.1038/s41467-019-12133-5)
Supplement: Supplementary file 1 — Supplementary Information [file 41467_2019_12133_MOESM1_ESM.pdf]

Resequencing 545 ginkgo genomes across the world reveals the evolutionary history of the living fossil

Zhao *et al*.

Supplementary Note 1. Sample collection and sequencing

The species and sample collection

Ginkgo (*Ginkgo biloba* L.) is one of the best-known and most distinctive trees worldwide[1,2]. It represents one of the four lineages of extant gymnosperm, i.e., cycads, ginkgo, conifers and gnetophytes, with cycads being its closest group[3,4]. As one of 16 different genera in Ginkgoatae during the early Permian (ca. 300 million years ago (mya)), the genus *Ginkgo* first appeared in the middle Jurassic (ca. 170 mya) and went extinct all over the world in the climate oscillation at the end of the Pliocene and the beginning of Pleistocene[5,6], with only a few relict populations survived in China[7-9]. Notably, this living fossil was redistributed out of China to different continents by humans and thus provides an inspiring example of how human can help a species survive and renew[7,8,10]. Ginkgo's resilience demonstrates an outstanding resistance or tolerance to both herbivores and pathogens, accounting in part for the longevity of the individual tree and also, in turn, for the longevity of this species[7,8,11].

In addition to extensive studies on population genetics, phylogeography and evolutionary history of this species[9,10,12-15], we have made great efforts to collect ginkgo samples around the world during recent decades. In the present study, we selected a total of 545 individual trees of *G. biloba* for resequencing. We sampled these trees either with a diameter at breast height (DBH) larger than 50 cm, corresponding to a minimum age of ~120 years[12], or with observed reproductive organs (Supplementary Table 1). Background information on populations such as habitats and historical references was also collected. These samples were collected during the last two decades from 51 populations across the world (Supplementary Fig. 1 and Supplementary Data 1), including 45 populations in China and 6 outside China (i.e., Japan, Korea, USA and Europe), covering most of the ever-known localities of old ginkgo trees. Although all the ginkgo trees outside China were reintroduced by humans within recent centuries, we tried to include samples in different continents to represent the entire gene pool of ginkgo around the world (Supplementary Data 1, Supplementary Table 1). A total of 87 big trees outside China were used in this study, including 21 from Korea, 34 from Japan, 16 from USA and 16 from Europe, which were pooled into six populations according to geographic

distributions and balanced population sizes (Supplementary Table 1). The 34 Japanese samples were divided into three populations accordingly. Although we treated the samples collected in Japan, Korea, USA and Europe as populations for convenience, all the populations represent many trees collected in different localities across the countries or regions (Supplementary Fig. 1 and Supplementary Table 1).

Given various sizes of sampled populations, we performed population genetic analysis by merging them into lineage to avoid the impact of various sizes of different populations on the analyses. The four Chinese lineages (i.e., EAST, SOUTH, NORTH, and SWEST) and an OVERSEAS lineage were defined according to their geographic distribution and phylogeographic patterns detected by the previous and present studies (see also in the main text).

DNA extraction and sequencing

Genomic DNA was extracted from silica gel-dried young leaves of 545 ginkgo trees using a standard cetyltrimethylammonium bromide (CTAB) method[17]. Genomic DNA was sheared into fragments with a length less than 1000 bp using ultrasound method on Covaris E220 (Covaris, Brighton, UK), followed by a size selection of 100–350 bp using AMPure XP beads (AGENCOURT). To ligate the sequencing adapter to the DNA fragments, we used T4 DNA Polymerase (Thermo Fisher Scientific, Waltham, MA, USA) and Klenow fragment enzyme (Thermo Fisher Scientific, Waltham, MA, USA) to repair the DNA fragments to get a blunt end; then a dATP was added to the 3'end to obtain a sticky end. The sequencing adapter with a dTTP was ligated to the double ends of the DNA fragments. Amplification of the ligation products was conducted for 8 cycles using Veriti™ Thermal Cycler (Thermo Fisher Scientific, Waltham, MA, USA).The standard circularization step required for BGISEQ-500 (MGI TECH, Shenzhen, China) was carried out and a single-strand circular DNA library (DNA Nanoballs, DNB) were prepared[18]. Finally, we sequenced all these samples using BGISEQ-500 with a pair-end read length of 50 bp or 100bp.

Raw reads filtering

To obtain high-quality reads for downstream analyses, raw data were filtered using SOAPnuke (ver. 1.5.4)[19] to remove the reads containing low quality bases greater than 10% (quality value < 10), unidentified bases (N) higher than 1%, and sequencing adapters with parameters -l 10 -q 0.1 -n 0.01 -Q 2. Then, we obtained clean sequencing reads with an average depth up to 6.1-fold, ranging from 4- to 10-fold for each sample (Supplementary Data 2 and Supplementary Fig. 2).

Supplementary Note 2. Identification of SNPs and quality control

SNP calling

Due to the large genome of ginkgo (10.6 Gb[12]) and huge amount of resequencing next-generation sequencing (NGS) reads, we used DRAGEN (http://www.edicogenome.com/) toolkit, a program featured by a high speed of mapping and SNPs calling in analyzing NGS data, to conduct reads mapping and SNP calling with parameters --enable-map-align=true, --enable-sort=true, --remove-duplicate=true, --vc-emit-ref-confidence=GVCF. Clean reads of FASTQ files were used for alignment, sorting, duplicates removing, and variant calling processing steps (Supplementary Fig. 13). About 99% of samples could be aligned to the reference genome with a mapping ratio more than 85% (Supplementary Data 1 and Supplementary Fig. 14**)**. Hidden Markov Model and Smith-Waterman Alignment of GATK 4.0 Haplotype variant caller[20] was packaged for the SNP calling of DRAGEN toolkit. Then, multiple individual SNP sets of gVCF files were jointly genotyped to generate a final population SNP set in VCF format.

Quality control of SNPs

We filtered the raw SNP set using a variant quality score recalibration (VQSR), which performs machine learning to identify annotation profiles of variants that are likely to be real and assigns a VQSLOD score to each variant. The filtering parameters of DRAGEN we used were QD < 2.0 || MQ < 30.0 || FS > 60.0. We further filtered out the SNPs with quality score less than 200 and identified a total of 161,040,296 high-quality SNPs

(Dataset 0). The majority of the SNPs showed high quality scores and small missing rates (Supplementary Fig. 15) with ~93% of SNPs located in the intergenic regions (Supplementary Fig. 3). Most of the distances between each two SNPs were less than 500 bp. We also observed an uneven density distribution of SNPs along the chromosomes as well as of both repetitive sequences and protein-coding genes (Supplementary Fig. 3).

To satisfy the requirements of various analyses (Supplementary Fig. 13), we complied additional four datasets derived from Dataset 0 using different filtering standards. It includes (1) Dataset 1: Q>200, MAF>=0.005, m<30% (No. SNPs: 133,646,277); (2) Dataset 2: Q>200, MAF>=0.005, m<30%, pruned LD>0.2 (No. SNPs: 23,056,642); (3) Dataset 3: Q>200, MAF>=0.01, m<30% (No. SNPs: 115,514,156); (4) Dataset 4: Q>200, MAF>=0.01, m<30%, pruned LD>0.2 (No. SNPs: 15,540,805)

Quality validation of SNPs

We randomly selected 14 samples from different populations and sequenced them using Illumina platform independently to assess potential biases resulted from different sequencing platforms. We called the SNPs using the same pipeline and parameters and compared the two SNP sets of these 14 samples generated by Illumina Hiseq2000 sequencing platform and BGISEQ-500 separately. We found that more than 98.6% of SNPs were shared by the two platforms in different filtering standards, suggesting the high consistence between the two sequencing platforms (Supplementary Table 2).

Supplementary Note 3. Chloroplast genome assembly, SNP calling and quality control

Raw sequencing data were filtered to obtain reads by mapping to the chloroplast sequence AB684440.1 of ginkgo[67] using BWA and SAMtools. Approximately 2.0 GB of the filtered data per sample was used to assemble the complete chloroplast genome using the GetOrganelle pipeline[68] and finally 446 chloroplast genomes were completely assembled. SNP calling and quality control for the chloroplast genomes are similar to those for the nuclear resequencing data. Briefly, paired-end reads were mapped to the reference genome

of ginkgo chloroplast using BWA mem. The mapping results were converted to BAM files using SAMtools. Picard package was used to sort BAM file, replace read groups and filter the PCR duplicates in sorted BAM file. SNPs were called using both GATK 4.0 and SAMtools. The low quality SNPs were filtered and were used to recalibrate base quality score of the BAM files. The resulting BAM files were used to detect the SNP using GATK 4.0 Haplotype Variant Caller[20]. GVCF files of all samples were combined to a VCF file that was recalibrated and filtered.

Supplementary Note 4. Population genetics analysis

Population genetic structure

We inferred population structuring and admixture of our global samples using ADMIXTURE (ver. 1.3.0)[21]. The most likely number of clusters (*K*=2 to 10), i.e., ancestral genetic components, was computed with five replicates and 10-fold cross-validation (CV) (Supplementary Fig. 4). Similarly, lowest CV values were observed for *K*=3 and 4. In combination with other population analyses including principal component analysis (PCA) and neighbor-joining tree, we considered *K*=4 as an appropriate model to subdivision of populations (Supplementary Fig. 5). For the plastome dataset, we successfully assembled 481 chloroplast genomes out of 545 accessions using MAFFT[69]. Haplotypes were defined for Chinese 397 chloroplast genomes based on the 67 annotated sequences (52 kb in length) on the GeSeq website[70]. The construction of the haplotype network was made using PopART[71]. The plastome NJ tree was reconstructed based on the SNPs called from 446 Chinese samples.

Genetic diversity at both species and lineage levels was estimated based on the dataset0. The nucleotide diversity π was calculated using the program VCFtools v0.1.13[53] and Watterson's estimator θ_W and Tajima's D were calculated using in-home perl script through scanning the whole genome with the non-overlapped 100 kb sliding window. For the chloroplast genome that is much smaller in size, the nucleotide diversity π was

calculated by sites using VCFTOOLS v0.1.13[53], and the number of segregating sites (*S*) and the number of haplotypes (*H*) were computed using the *R* script 'PopGenome'[72].

Principal component analysis

Principal component analysis (PCA) was conducted to study the relatedness and clustering among populations or samples. The top 10 PCs of the variance-standardized relationship matrix were extracted using PLINK (ver. 1.90)[22] with --pca parameter based on Dataset2. Then, the figures of first three PCs were drawn using Origin® 2018 (Origin Lab, Northampton, MA). The PCA analysis of the Chinese samples was performed to investigate the population genetic patterns in China (Supplementary Fig. 5). An additional PCA for 545 global samples was focused on the origin of non-China samples (Supplementary Fig. 11).

Phylogenetic analysis

To quantify the relatedness between individuals, pairwise identity-by-state (IBS) genetic distance matrix of was calculated for 545 samples using PLINK[23] (ver. 1.90) with parameter -distance 1-ibs based on the Dataset 2. We constructed neighbor-joining (NJ) phylogenetic tree using MEGA (ver. 4.0)[24] based on the distance matrix (Supplementary Figs. 6 and 7). The resulting individual NJ tree demonstrates a congruent population structure pattern with ADMIXTURE (Supplementary Fig. 4). A population-level NJ tree was also constructed based on the genome-wide weighted F_{ST} pairwise distance matrix among all populations with a minimal size of 2 calculated from Dataset3 using PLINK (ver. 1.90). The inter-population NJ tree showed that populations from EAST, SOUTH and SWEST well clustered while those from NORTH (in grey) were scattered in the former three lineages (Supplementary Fig. 5). Similarly, phylogenetic analysis of the chloroplast genomes was conducted for Chinese ginkgo trees.

Supplementary Note 5. Demographic history

Calibration of mutation rate using seed plant phylogeny and fossil constraint

The phylogenetic tree was constructed by the single copy orthologs of *Ginkgo biloba*[12], *Cycas revoluta* (transcriptomes NGS reads from NCBI SRA database: SRR1525778), *Picea abies*[25], *Gnetum parvifolium*[26], *Amborella trichopoda*[27] and *Populus trichocarpa*[28] to estimate the mutation rate of the ginkgo genome. Because the genome of *Cycas revoluta* has not been reported yet, we used the assembled sequences of the transcriptome reads (SRR1525778) downloaded from NCBI. We assembled the reads with Trinity-2.0.6[29] and filtered the resulting sequence with Seqclean preceding clustering with TGICL[30] to get the unique transcripts. Transdecoder (https://github.com/TransDecoder/TransDecoder) was used to detect the ORF structure with the default parameters. First, we identified the single-copy orthologues of those six species. For gene family analysis, BLASTP (E-value ≤ 1.0e-05) was used to compare the protein sequences between ginkgo and the other five species. The paralogs and orthologues were clustered with OrthoMCL[30]. The single-copy orthologs in the cluster were used to construct the phylogenetic tree with PhyML (-m F84, -a invgamma, -b -2). The divergence time between ginkgo and other five species was estimated using MCMCTREE[31] referring to the calibration time (Supplementary Fig. 16). Finally, the estimated mutation rate of the ginkgo sequence was about 0.67×10^{-9} (per base per year), similar to the synonymous mutation rate of 0.68×10^{-9} between gymnosperm *Picea sitchensis* and *Pinus taeda*[32]. The resulting estimates for the crown ages of ginkgo-cycads, gymnosperm and seed plants are congruent with previous studies[32].

Inference of demographic history using PSMC

To infer historical dynamics of effective population size and timing of ginkgo linkages/groups, we sequenced 8 samples from four linkages in China (Supplementary Table 11) with a high sequencing depth (~30-fold) to ensure the quality of PSMC analysis[33]. The whole genome diploid consensus sequences for each sample were generated by samtools and bcftools with parameter C50. Sites with sequencing depth <10 and >100 (vcfutils.pl vcf2fq -d 10 -D 100) were removed to reduce the false results. The parameter of PSMC psmc -N25 -t15 -r5 -p 4+25*2+4+6 was used to infer the history effective population size. The estimated generation time and mutation rate were set to 20

and 0.67e-9, respectively. In addition, we carried out the estimation of another two different generation time including 40 and 80 years, revealing high consistence (Supplementary Figs. 10, 16 and 17). In detail, we performed the PSMC analysis using the following commands: (1) samtools mpileup -C50 -ufref.fastasample.bam | bcftools view -c - | vcfutils.pl vcf2fq -d 10 -D 100 |gzip ->diploid.fq.gz; (2) utils/fq2psmcfa -q20 diploid.fq.gz >diploid.psmcfa; (3) psmc -N25 -t15 -r5 -p 4+25*2+4+6 -o diploid.psmcdiploid.psmcfa; and (4) utils/psmc plot.pl -u 1.33e-8 -g 20 -p diploid diploid.psmc.

Inference of demographic history using *fastsimcoal2*

The more recent demographic histories of the major lineages (i.e., EAST, SOUTH, NORTH, SWEST) was reconstructed using *fastsimcoal2*[34]. According to the PCA and ADMIXTURE results, we tested 28 models that considered a combination of two migration matrices (total absence of post-divergence gene flow and a full migration matrix of asymmetric gene flow), five alternative scenarios of lineage divergence/formation and three possibilities of ancestry (a putative common ancestor, the SWEST and EAST lineages) (Supplementary Fig. 17). The selection of possible models were based on the ADMIXTURE and PCA results that suggested four lineages (i.e., EAST, SOUTH, NORTH, and SWEST) and showed the deepest divergence resulting in the SWEST lineage and the closest relationship between the EAST and SOUTH lineages. Between-population gene flow was also considered due to the presence of the individuals with admixed genetic components. We estimated the composite likelihood of the observed data given a specified model using the site frequency spectrum (SFS)[35]. For the simulations, 40 individuals were randomly selected from each of the four lineages (i.e., EAST, SOUTH, NORTH, and SWEST) with emphasis on the major populations. More than 1,742,493 noncoding SNPs were called to a folded joint SFS considering a single SNP per locus to avoid the effects of lineage disequilibrium. We assumed a mutation rate of 0.67e-9 per site per year and a generation time of 20 years in ginkgo when converting estimates to units of years and individuals.

Two-dimensional joint SFS (2D-SFS) was constructed from posterior probabilities of sample allele frequencies using easySFS.py (https://github.com/isaacovercast/easySFS). Each model was run 100 replications considering 100,000 simulations for the calculation of the composite likelihood, and 10–40 expectation-conditional maximization (ECM) cycles. Model comparison was based on the maximum value of likelihood over the 50 independent runs using the Akaike information criterion and Akaike's weight of evidence[34]. The model with the maximum Akaike's weight value was chosen as the optimal one. Finally, we calculated confidence intervals of parameter estimates from 100 parametric bootstrap replicates by simulating SFS from the maximum composite likelihood estimates and re-estimating parameters each time[36,37].

Model scoring supported the most likely scenario (Model 3-the putative ancestor, Supplementary Table 5) of the initial split of SWEST from the putative ancestor and followed divergence between EAST and SOUTH preceding the admixture origin of NORTH between SWEST and SOUTH. Detailed estimates of demographic parameters, i.e., divergence time, gene flow, effective population sizes and the corresponding 95% confidence intervals (CIs) obtained through bootstrapping are summarized in Supplementary Table 5. The first divergence leading to the origin of the lineage SWEST occurred as early as 515,780 years ago (T_1, CI: 489,340-613,980). The subsequent split between the lineages SOUTH and EAST was estimated to take place 318,120 years ago (T_2, CI: 294,180-329,080). The admixed NORTH lineage formed about 139,260 years ago (T_3, CI: 82,160-141,180) with the genetic components from SWEST (28.25 %, CI: 26.63%–34.77%) and SOUTH (71.55%, CI: 65.23%–73.37%). All the four lineages showed comparable contemporary effective population sizes (*N*e), which are smaller than that of their common ancestor (52,750, CI: 17,438-86493 (Fig. 2b). In contrast, the SWEST lineage remains a strikingly small population size of 669 (CI: 563–805). The derived NORTH group experienced exponential growth, the effective population sizes reached as large as 212,106 (CI: 192,638–214,744). Interestingly, the best model didn't detect obvious gene flow between the SOUTH, EAST and SWEST lineages except the admixture forming the NORTH lineage.

Isolation by distance model

To infer relationship between samples, pairwise genetic distance of identity by state (IBS) was calculated using PLINK (ver. 1.9). The histogram of IBS was used to display the IBS distribution. Pairs with low genetic distances (IBS < 0.03, 0.109% of all pairs or IBS < 0.07, 3.189% of all pairs) may reflect the recent dispersal or introduction. The connection lines between samples were drawn on the map of both China and world.

Supplementary Note 6. Species distribution modeling

Species distribution modeling (SDM) is a widely used method to explore the impacts of climate on species distribution and to detect the climate variables that contribute to the species distribution[40-42]. We generated SDM for ginkgo trees using the software MAXENT v3.3.1[42,43]. The occurrence records of sex-matured ginkgo trees were retrieved from the literature[44], the Ginkgo Pages (http://www.xs4all.nl/~kwanten/), and our own field investigations. It should be noted that the occurrence data were selected according to the criterion of observed blooming and/or fruiting organs in ginkgo trees grown outdoors. Such a phenomenon suggests a likely reproduction of ginkgo trees under the local environments. We did not attempt to classify them into natural or cultivated populations since our aim is to simulate the potential ranges suitable for the survival and reproduction of ginkgo trees regardless of the dispersals realized by either wild animals or humans. To reduce sampling bias in the modeling[73,74], we removed the occurrence records with an interval distance less than 5 km and finally obtained a total of 146 records that covered the entire distribution areas in China.

The environmental layers of 19 biologically meaningful climate variables (BIO1-BIO19) (Supplementary Table 7) at present (average for the years 1970-2000), during the last glacial maximum (LGM: c. 21 thousand years before present (kyr BP)), and the last interglacial (LIG: c. 115-130 kyr BP) were downloaded from the WorldClim database website (http://www.worldclim.org/)[45]. All variables were evaluated for their relative contributions to models using the jackknife test[75,76]. Pairwise Pearson correlation coefficients were calculated for the 19 variables to test the effect of multicollinearity

among predictor variables, which could result in the over-fitting of models[77,78]. Seven variables with relatively lower Pearson correlation coefficients (r^2< 0.80)[76] and relatively higher contributions to the model (>0.3) were employed in the SDM analyses, i.e., mean temperature of the coldest quarter (BIO11), temperature seasonality (BIO4), mean temperature of the wettest quarter (BIO8), precipitation of the driest month (BIO14), precipitation seasonality (BIO15), precipitation of the warmest quarter (BIO18), and mean diurnal range (BIO2).. The distribution predictions for LGM and at present were performed under the CCSM4 model layers with 2.5-arc minutes resolution[45] while the prediction for LIG was projected using the data from Otto-Bliesner[46] with 30-arc sec resolution. To obtain the climate data at the same resolution, the environmental layers for LIG were re-sampled using ArcGIS (http://www.esri.com/). The analysis was run with 100 replicates via the subsample method, followed by 70%/30% train/test partitions of occurrence points[41]. As a measure of the accuracy of the SDMs, we used the threshold independent and prevalence insensitive parameter, area under the curve (AUC) of the receiver operating characteristic (ROC) plot produced by Maxent[47].

Supplementary Note 7. Analysis of footprints of natural selection

Detection of genomic regions with signature of selection

Our analysis of population genetic structure based on 545 ginkgo genomes detected three ancient refugia in the eastern, southern, and southwestern China and indicated high genetic differentiation between the EAST and SWEST lineages (Fig. 1). Given the EAST and SWEST lineages showed different habitat preferences (Supplementary Table **7**), we detected genome regions under selection for the EAST and SWEST lineages to explore the genomic basis of local adaption in the eastern and southwestern China, respectively. The two groups (each consisting of 58 individuals) contain main genetic components of ginkgo and grow in typical habitats of the EAST and SWEST lineages, referred to as EAST-g and SWEST-g, respectively. The EAST group (EAST-g) consists of 9 subpopulations at TM while the SWEST group (SWEST-g) includes 2 subpopulations at WC and JF (Fig. 1a and Supplementary Table 8). To obtain SNPs from the groups, we extracted data of the 116 individuals from the dataset0 and filtered out the SNPs of

monomorphic or missing sites within EAST-g or/and SWEST-g, respectively (Supplementary Table 8). Based on the filtered data, we detected genomic regions with signatures of selection using two approaches.

Generally, genomic regions under purifying selection within either EAST-g or SWEST-g may show a lower diversity and increased fixation index between them[48-50]. Thus, we first used population genetics summary statistics (H_E and F_{ST}) to identify genomic regions with signatures of purifying selection for the two groups separately. We calculated the heterozygosity following the method described in[51] and the fixation index (F_{ST})[52] using the program VCFTOOLS v0.1.13[53] in 100kb sliding windows along all chromosomes. We Z-transformed the resultant distribution of H_E and F_{ST} values and applied a threshold of $Z(H_E) < -2.58$ and $Z(F_{ST}) > 2.58$ for identifying putative selective regions because windows below this threshold represent a statistical confidence interval of 99% (Supplementary Fig. 18).. Because a recently fixed beneficial mutation will eliminate the genetic variation at the nearby locus linked, resulting in selective sweep[54], we thus performed a likelihood-based program SweeD (version 3.1)[54,55] to detect putative regions of positive selection by scanning the genome for signals of hard (fixed) selective sweeps along all chromosomes in the two groups, respectively. This program identifies regions that are significantly deviations from neutral SFS by implementing composite likelihood ratio (CLR) statistics and performs better with genome-wide SNP data than similar programs. We estimated SFS of the EAST-g and SWEST-g separately by SweeD (using the MPFR library) based on the PSMC results. The top 1% of CLR was taken as the cutoff and the neighbor sweep targets were merged into the putative sweep regions. Using this approach, we identify 903 sweeping regions with signatures of selection sweep in each of the two groups (Supplementary Fig. 19, Supplementary Data 6). Together, we obtained a total of 910 and 949 putative regions with signature of selection, containing 643 and 504 candidate genes, in EAST-g and SWEST-g, respectively (Supplementary Data 7).

Pathways and genes involving local adaptation

We performed gene ontology (GO) enrichment analysis for those candidate genes in EAST-g (643) and SWEST-g (504) (Supplementary Data 7) using perl script[56]. We

obtained 14 and 17 significantly enriched terms ($P<0.05$) in EAST-g and SWEST-g, respectively (Supplementary Table 9), including many terms related to adaptations such as responses to various biotic and abiotic stresses. Of these terms, two (GO:0006950 and GO:0006952) involve defense responses and stresses in EAST-g, and another two (GO:0051716 and GO:0010038) involve response to stimulus and metal ion in SWEST-g (Supplementary Table 9). We further performed homology analyses of the 5 and 20 genes in EAST-g and SWEST-g, respectively, which were identified by both approaches mentioned above (Supplementary Table 10). In EAST-g, we found a candidate gene (Gb_18615) that has a polyphosphoinositide binding domain and is believed to play a key role in inositol lipid biology[57]. This gene is homologous to VIP1 and VIP2 in *Arabidopsis* and involves jasmonic acid signaling, insects and fungi defense[58,59]. In SWESTg, we found five candidate genes that may contribute to the adaption of ginkgo to local environments, including four NBS-LRR (nucleotide-binding site and leucine-rich repeat domains) family genes (Gb_11158, Gb_26523, Gb_36252 and Gb_12753) (Supplementary Table 10). This family of proteins are encoded by hundreds of diverse genes per genome and have been reported to bind to pathogen effector proteins to activate multiple defense signal transduction in plant[60-62]. Another gene (Gb_12751) has the LRR domain and is homologous to RPK1 and RPK2 (receptor-like protein kinase) in *Arabidopsis* and is the key player in ABA signaling and responses to abiotic stress such as dehydration, low temperature and high salt[63-66]. We also found three homologous genes (MA_13025g0010, MA_10427820g0020 and MA_129592g0010) of Gb_12751 in *Picea abies* (http://congenie.org), which were all highly expressed in needles and pineapple galls. Additional analysis of these genes will be worthwhile in future.

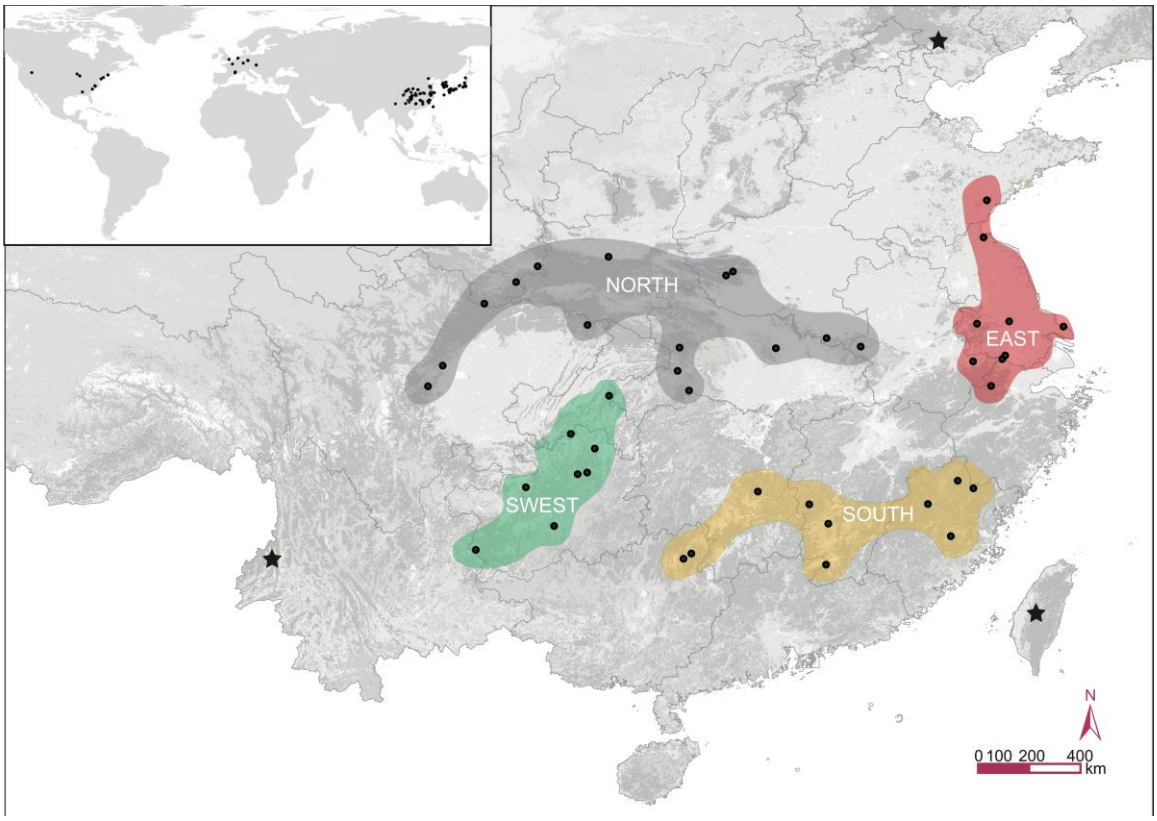

Supplementary Fig. 1. Geographical distribution of ginkgo trees collected in this study. Sampling locations in China are enlarged in the main picture, with three lineages in the putative refugia in eastern, southern and southwestern China (i.e., EAST, SOUTH and SWEST) and one admixed lineage in northern China (i.e., NORTH) indicted by shades in different colors. Three localities with stars indicate the populations that were proposed to be dispersed by humans. Insert: the sampling locations around the world, including China and 8 other countries in different continents (Supplementary Table 1). The map image, derived from ArcGIS Online maps, is the intellectual property of Esri and is used herein under license.

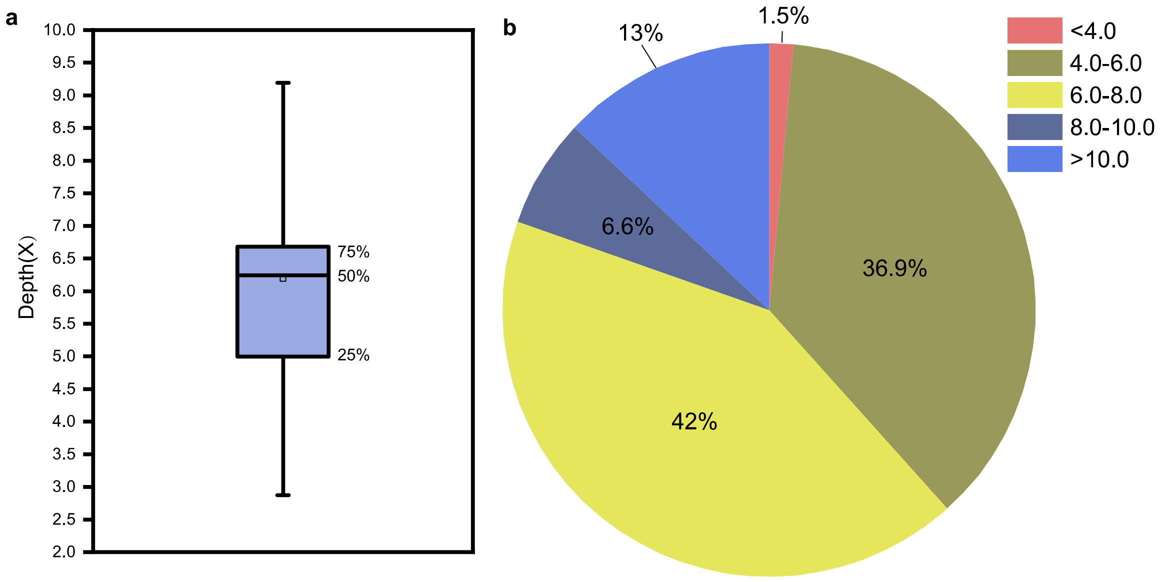

Supplementary Fig. 2. Distribution of sequencing depth of 545 ginkgo trees. (a) Boxplot of sequencing depth. The square in the boxplot represents the average depth (6.1x). The upper limit is calculated by the formula: Q3+1.5×(Q3 – Q1) = 9.2x, and the lower limit is calculated by the formula: Q1 − 1.5×(Q3 – Q1) = 2.9x. **(b)** Pie chart of sequencing depth. The number in each sector of the pie charts represents the percentage of whole samples. Source data are provided in a Source Data file.

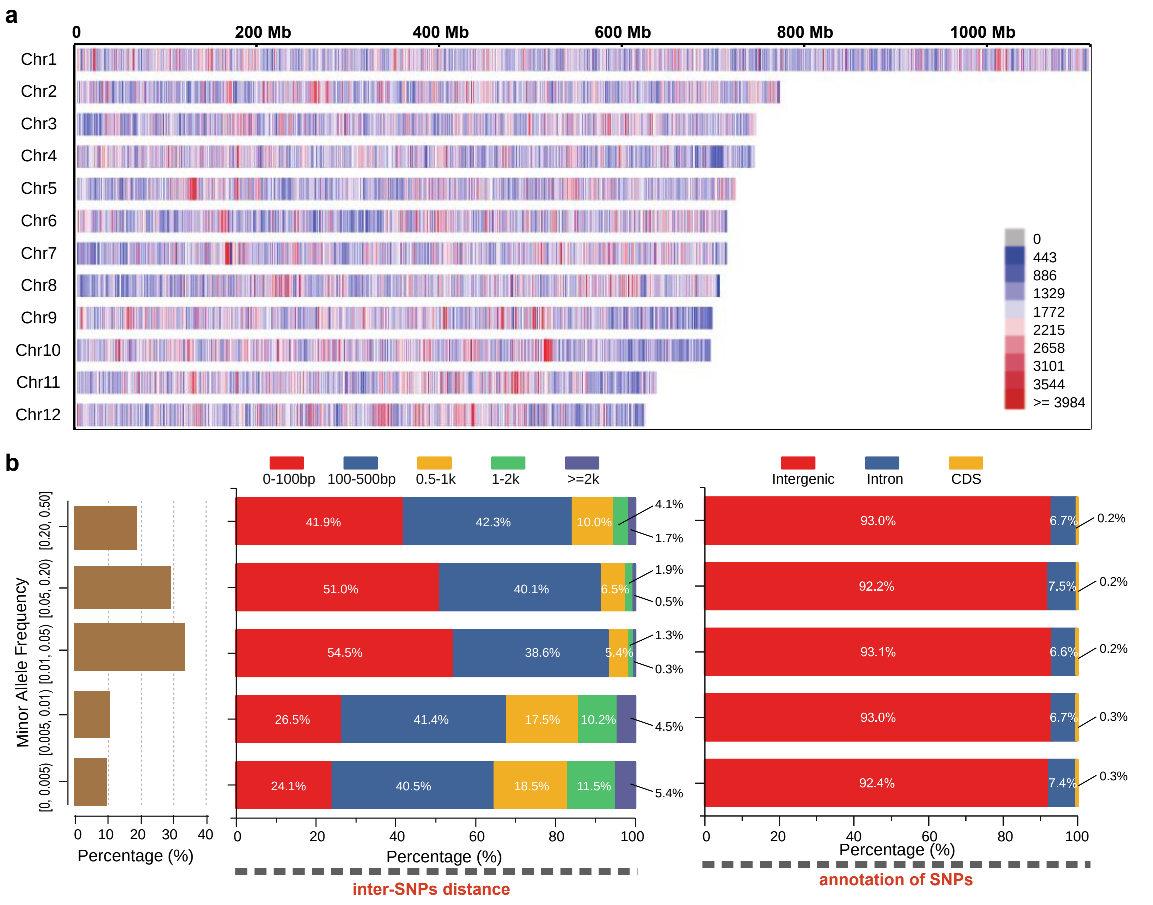

Supplementary Fig. 3. The density and distribution of SNPs of dataset 0. **(a)** SNPs density along chromosomes in 100 kb windows. Colors indicate the number of SNPs per 100 kb window. Gaps in the assembly are shown in gray. (**b**) Distribution of minor allele frequency, neighbor SNPs distance and function annotation for SNPs in ginkgo populations. Colors indicate the distance of neighbor SNPs or putative SNPs function. The source data of Supplementary Fig. 3b are provided in a Source Data file.

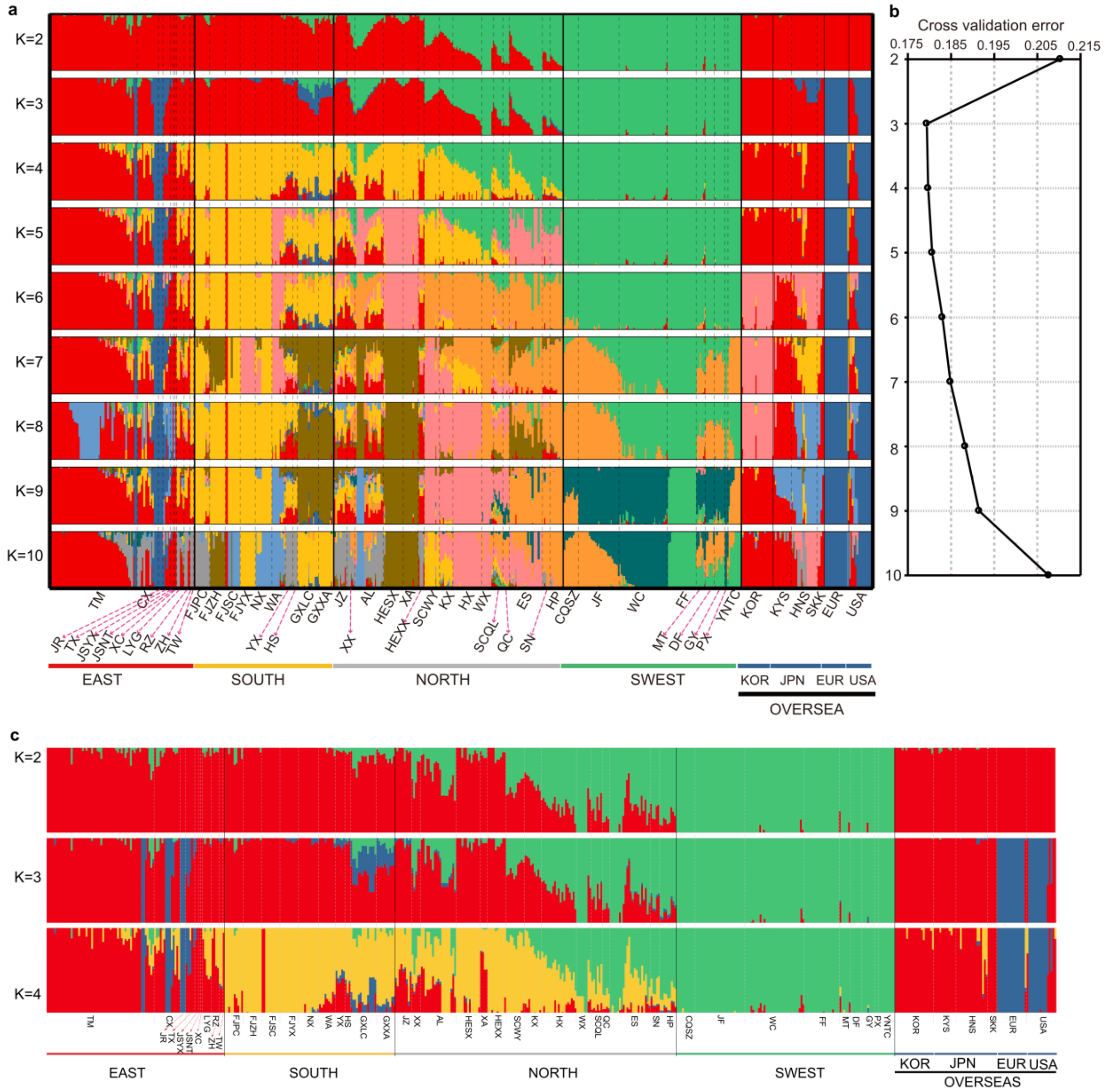

Supplementary Fig. 4. Population structure analysis of sequenced ginkgo based on SNPs of dataset 2. (a) ADMIXTURE results (*K*=2 to 10). The y axis shows the highest likelihood replicate for each *K* value and x axis indicates the populations. (**b**) Cross validation (CV) error value for different model *K*. (**c**) ADMIXTURE results (*K*=2 to 4) obtained based on the draft genome.

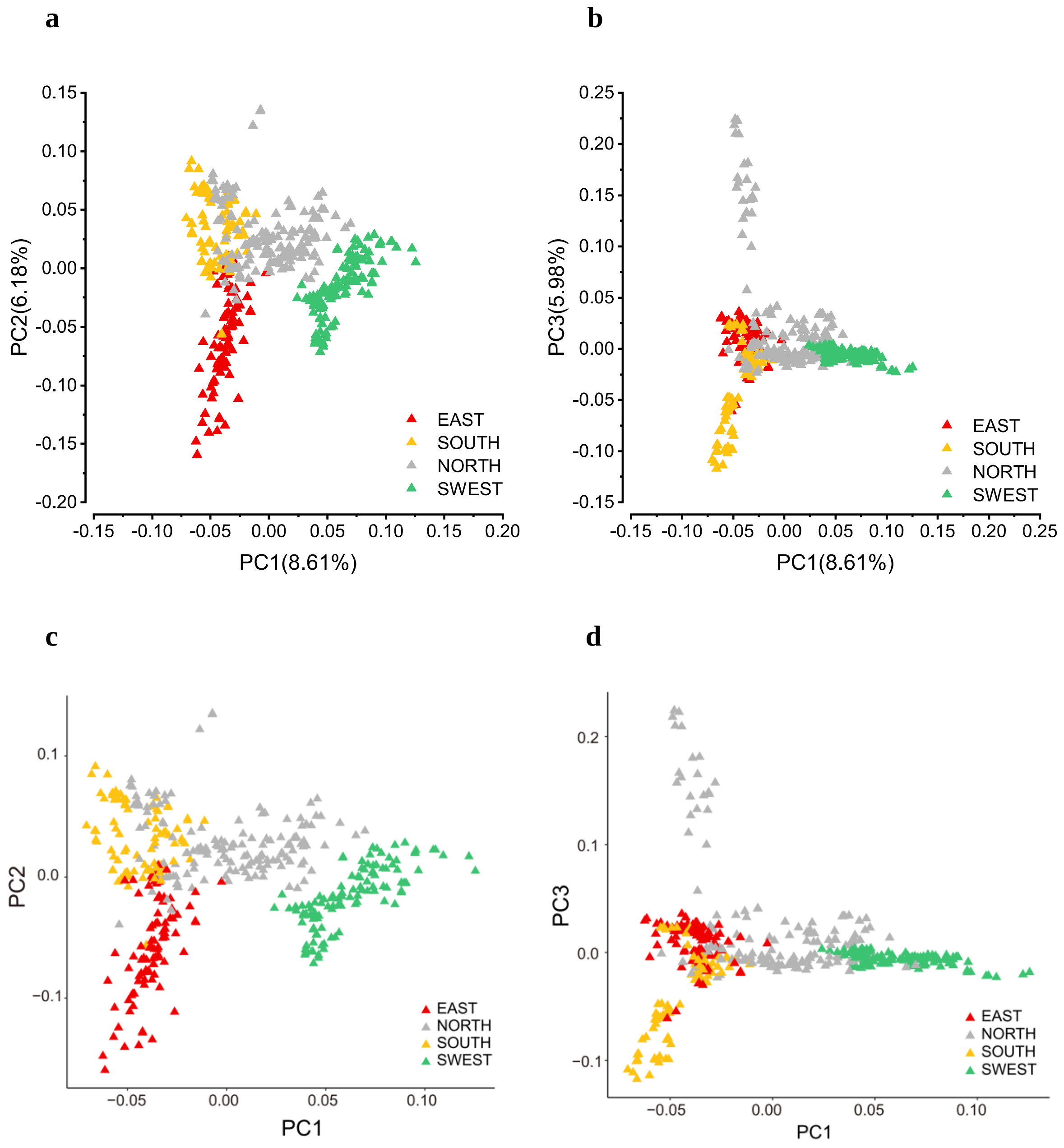

Supplementary Fig. 5. Principal component analysis plot of 458 ginkgo samples from China. **(a)** the first two principal components. **(b)** the first and third principal components. Colored triangles represent individuals of four different lineages. (**c, d**) PCA results conducted based on the draft genome. The source data of Supplementary Fig. 5a and 5b are provided in a Source Data file.

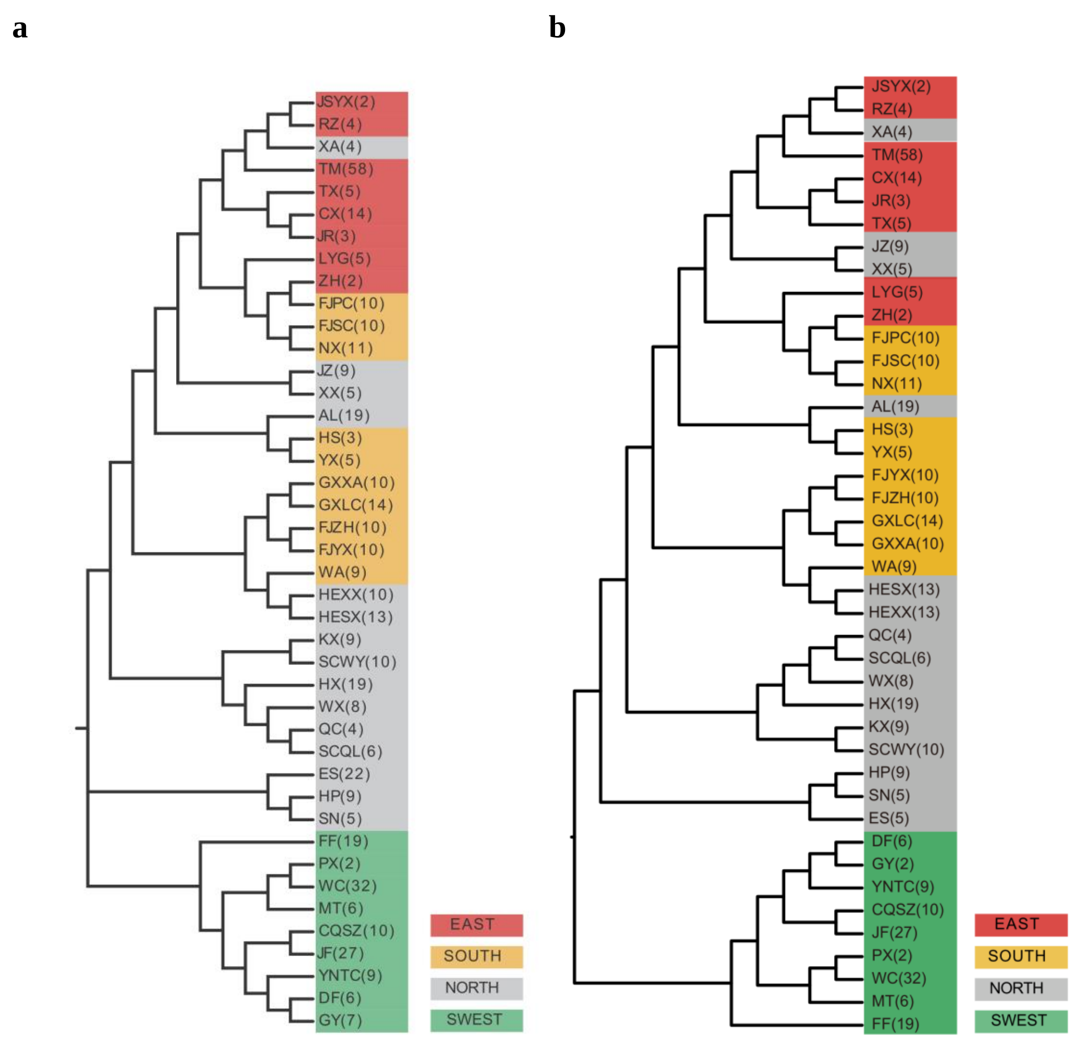

Supplementary Fig. 6. Neighbor-joining tree of Chinese populations of ginkgo based on F_{ST} values of resequencing data. Analyses were conducted using SNPs based on the updated genome (**a**) and the draft genome (**b**). The numbers in brackets following population codes indicate sample size of the populations (at least two samples). Populations from the four lineages (i.e., EAST, SOUTH, NORTH and SWEST) were highlighted in different colors. The source data of Supplementary Fig. 6a are provided in a Source Data file.

a

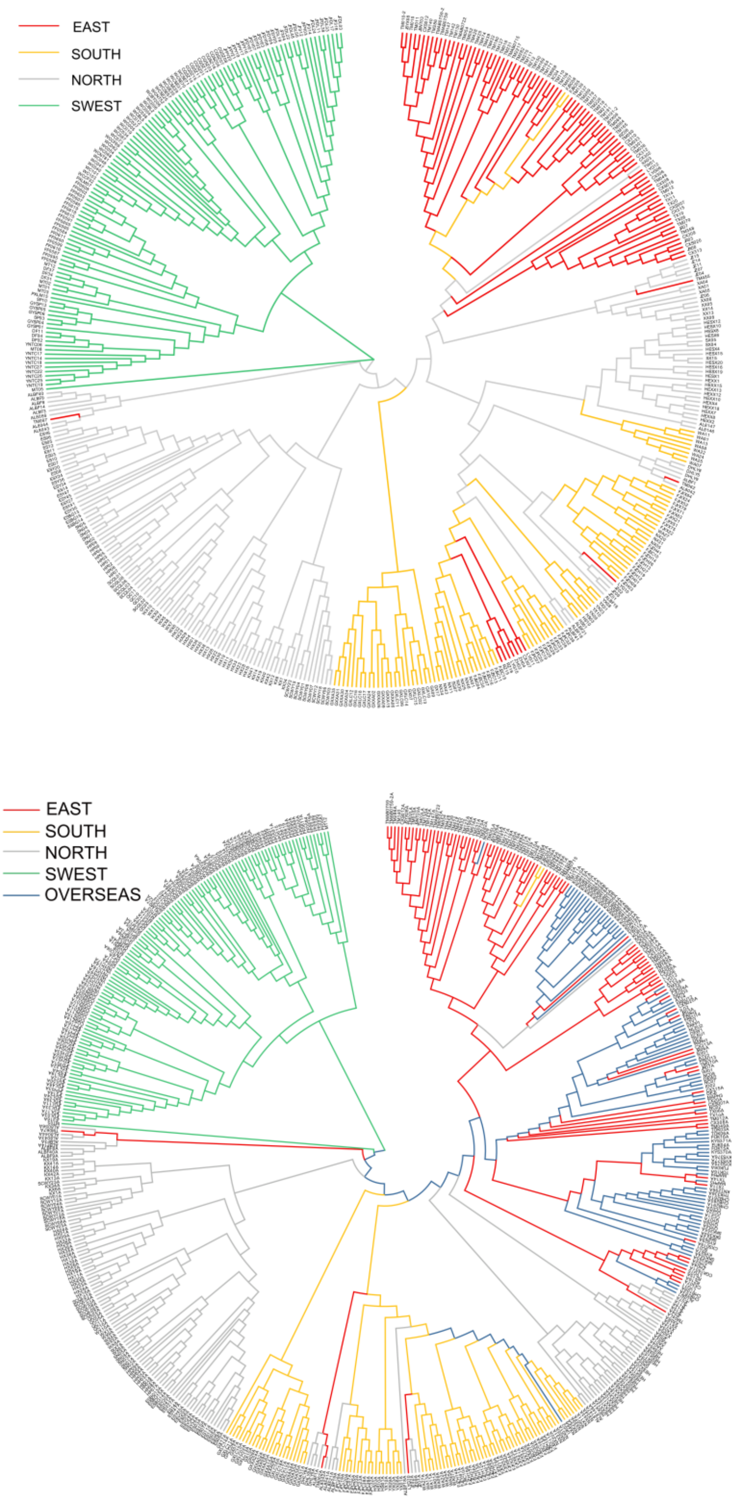

Supplementary Fig. 7. Neighbor-joining tree of ginkgo samples generatedbased on resequencing data. (**a**) Analysis of Chinese samples using SNPs based on the updated genome. (**b**) Analysis of world samples using SNPs based on the draft genome. Colored lines represent individuals of different lineages.

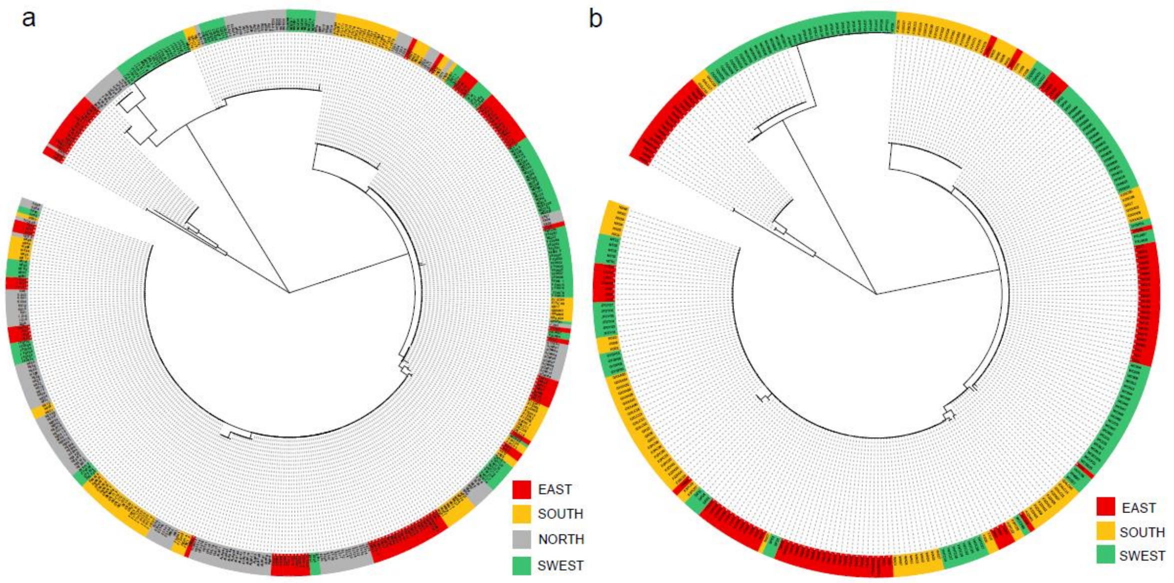

Supplementary Fig. 8. NJ trees of ginkgo samples from China based on whole-chloroplast genome sequences. (**a**) all 446 samples across China and (**b**) 298 samples from the EAST, SOUTH, and SWEST lineages. Colored lines represent individuals of different lineages.

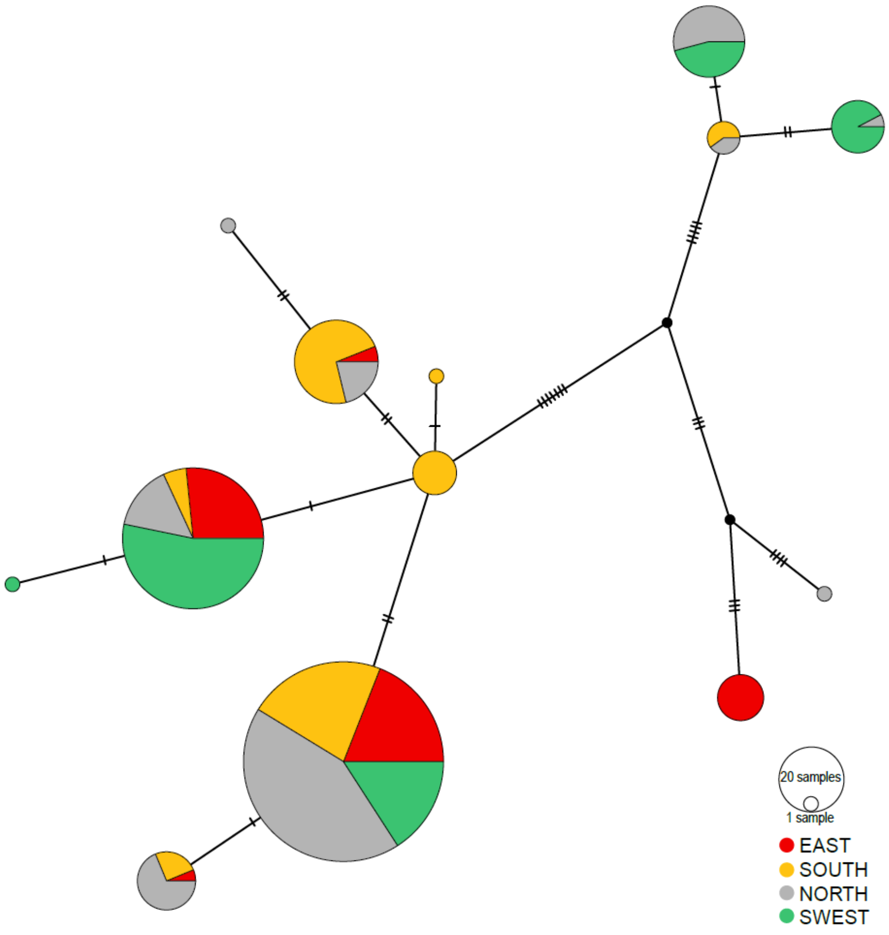

Supplementary Fig. 9. Network of 17 chloroplast haplotypes for Chinese ginkgo trees. Size of pie charts is proportional to the number of individuals assigned to the respective haplotype, with different colors representing lineages of origin. All haplotypes were clustered into three major and distinct groups, corresponding to three major clades revealed by the chloroplast NJ tree.

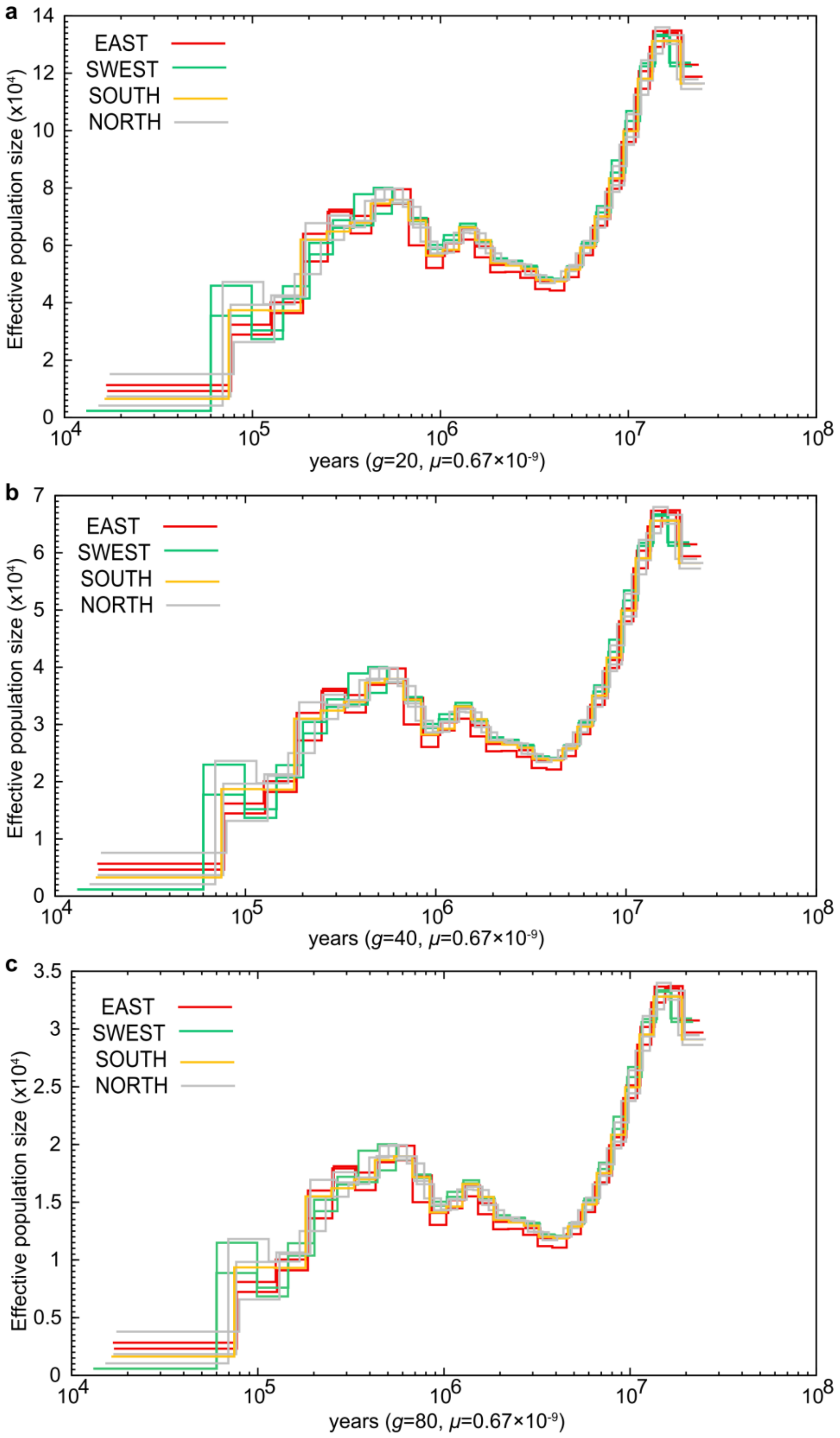

Supplementary Fig. 10. Inference of historical effective population sizes for ginkgo. The inferences were performed with the generation time being 20 (a), 40 (b), and 80 (c) years.

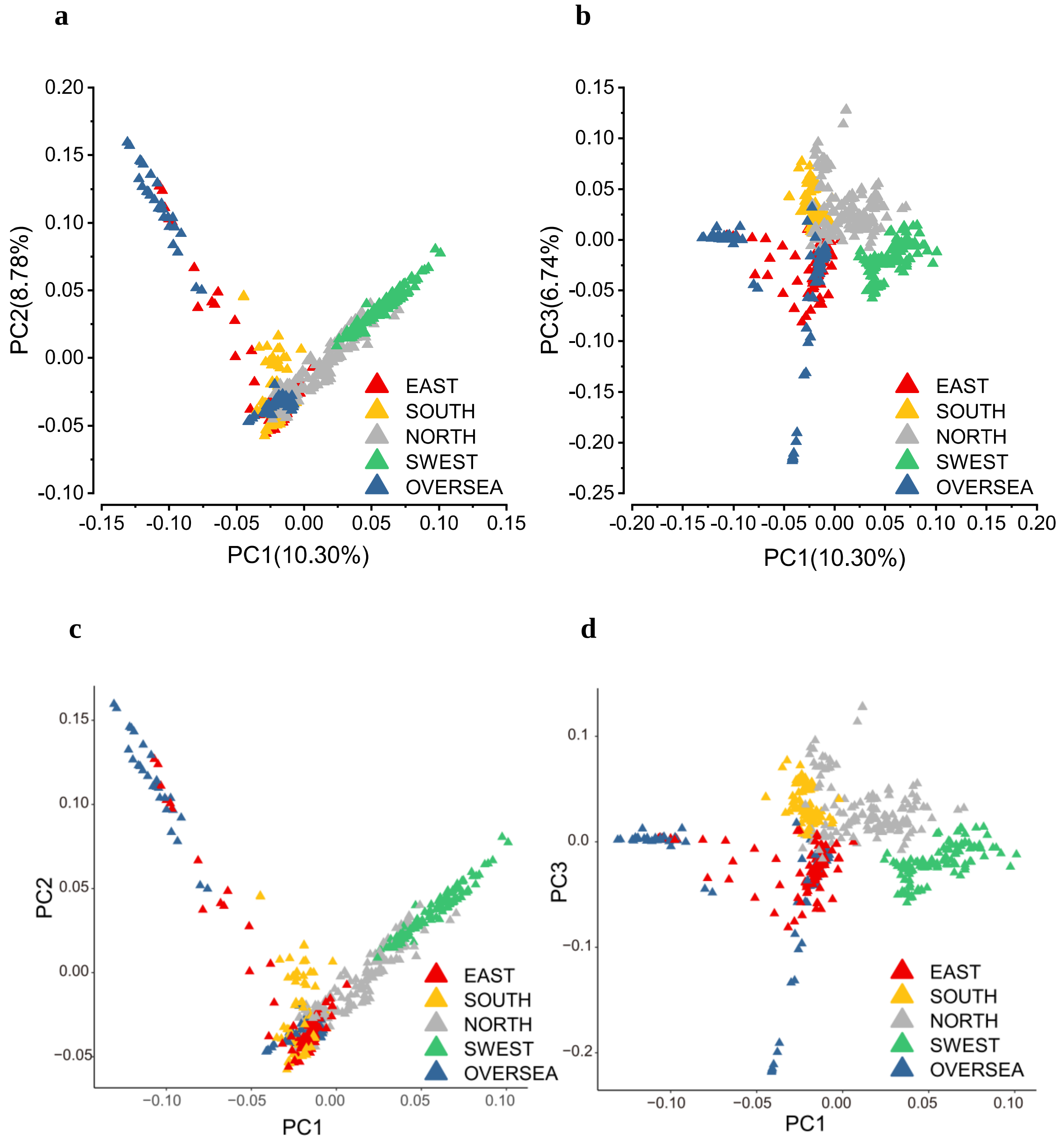

Supplementary Fig. 11. Principal component analysis plot of 545 ginkgo samples around the world. **(a)** the first two principal components. **(b)** the first and third principal components. Solid triangles in colors represent individuals of five different lineages. (**c, d**) PCA results conducted based on the draft genome. The source data of Supplementary Fig. 11a and 11b are provided in a Source Data file.

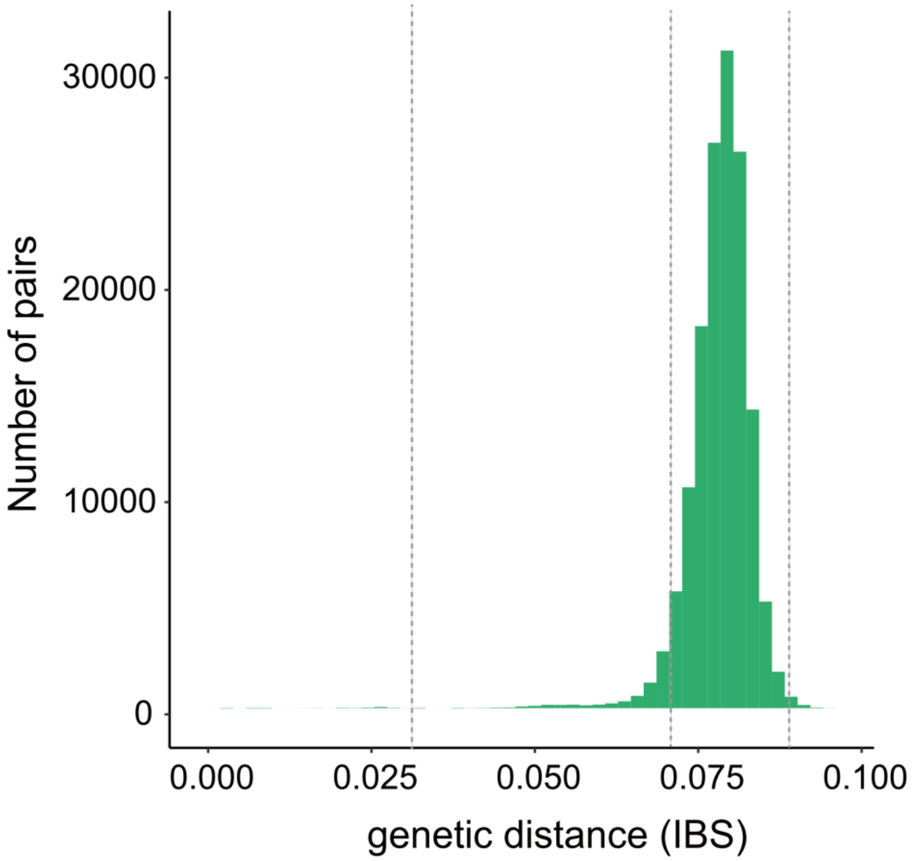

Supplementary Fig. 12. Distribution of pairwise identity-by-state genetic distances calculated using SNPs based on the draft genome. IBS: identity-by-state.

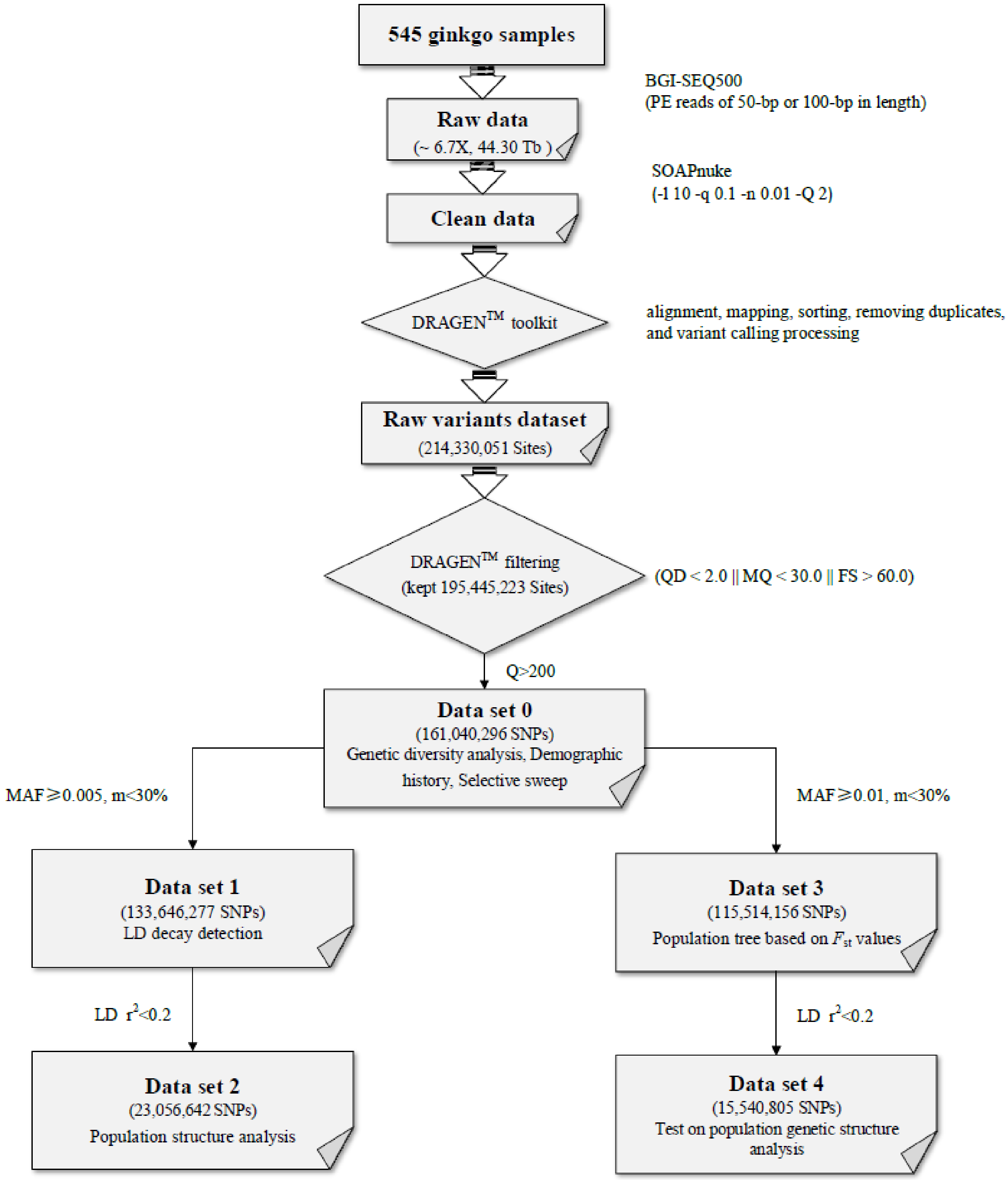

Supplementary Fig. 13. Workflow of single-nucleotide polymorphisms detection and filtering for ginkgo data sets.

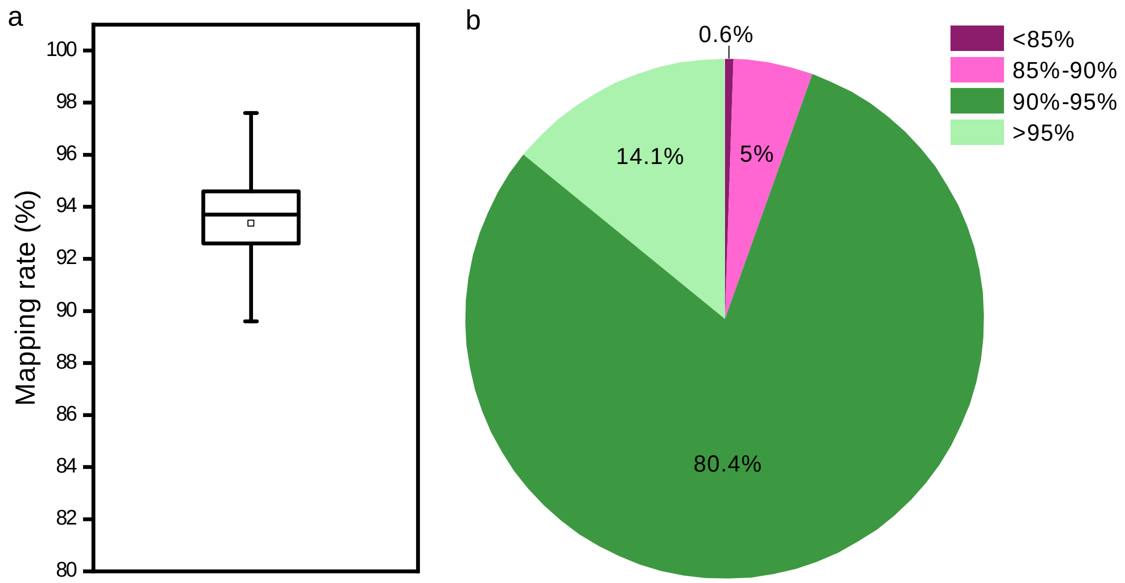

Supplementary Fig. 14. Mapping rate of 545 ginkgo individuals. (a) Boxplot of mapping rate. The square in the boxplot represents the average mapping (93.8 %). The upper and lower limits were calculated by the formula: Q3+1.5×(Q3–Q1) = 97.6% and Q1–1.5×(Q3–Q1) = 89.6%, respectively. **(b)** Pie chart of SNPs mapping rate. The number in each sector of the pie chart represents the percentage accounting for the whole SNPs. Source data are provided in a Source Data file.

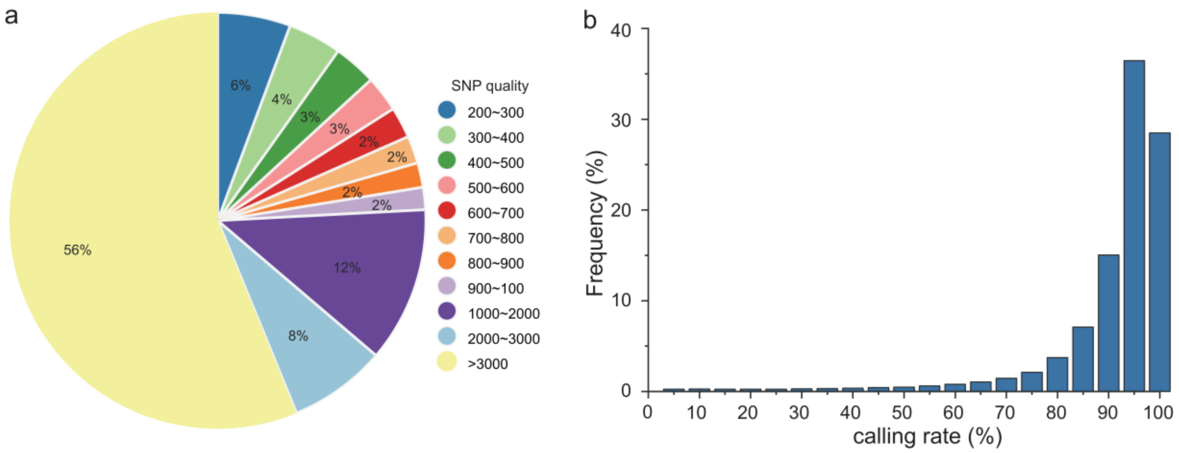

Supplementary Fig. 15. Distribution of SNPs quality and calling rate of dataset 0. (a) Distribution of SNPs quality. The number in each sector of the pie chart represents the percentage of SNPs. **(b)** Distribution of SNPs calling rate at the site level. Source data are provided in a Source Data file.

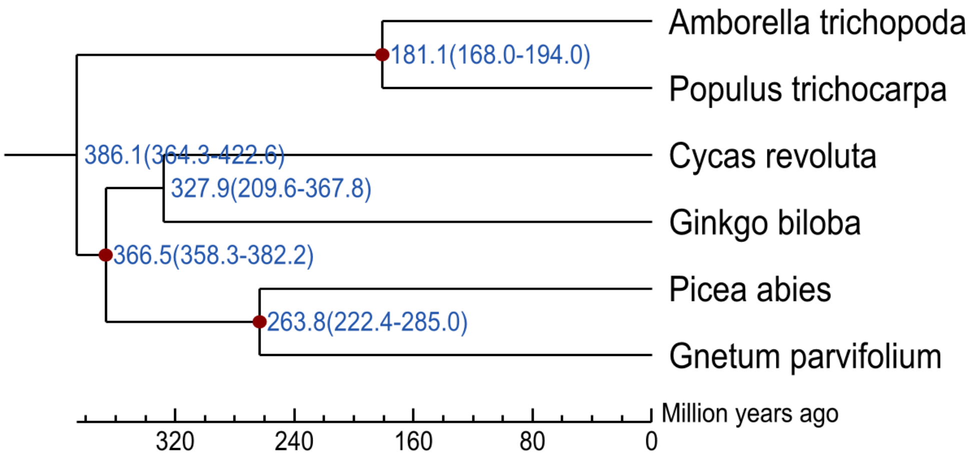

Supplementary Fig. 16. Time-calibrated phylogenetic tree of six seed plants. The tree was calibrated with *Amborella trichopoda-Populus trichocarpa* split of time range 168-194 mya, *Picea abies-Gnetum parvifolium* split of time range 218-283 mya. The two calibrated times was obtained from TreeTime[38]. The time of gymnosperms' common ancestor of 359-385 mya was also used as one calibration point[39]. Those calibrated times were pointed in red dots.

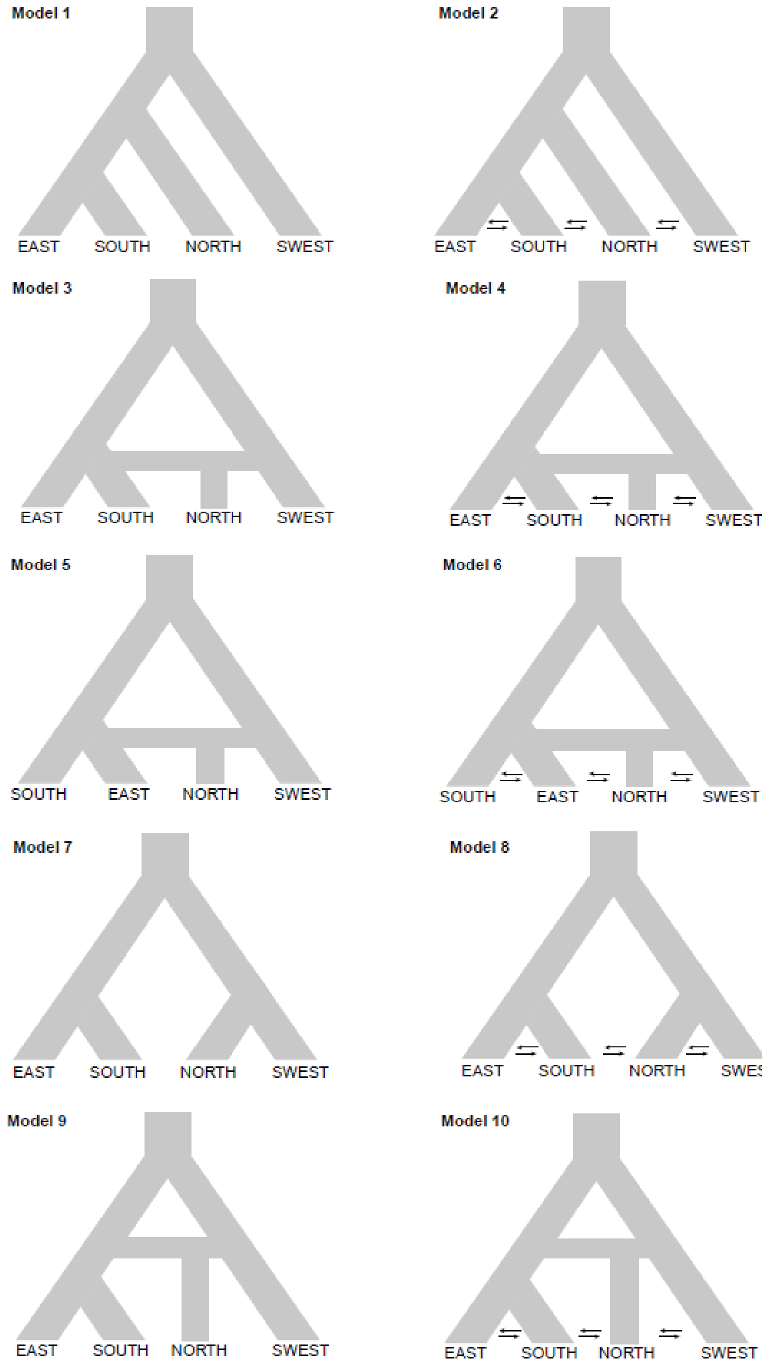

Supplementary Fig. 17. Ten basic demographic models analyzed with *fastsimcoal2*. 40 individuals were selected from each of the four lineages/groups identified by the ADMIXTURE and PCA analyses (i.e., the EAST, SOUTH, NORTH and SWEST lineages). Between-population gene flow was indicated by the arrows (Model 1~10). Based on the ten basic models, two more possibilities of ancestor were also considered, i.e., EAST (Model1a~10a) and SWEST (Model1b~10b).

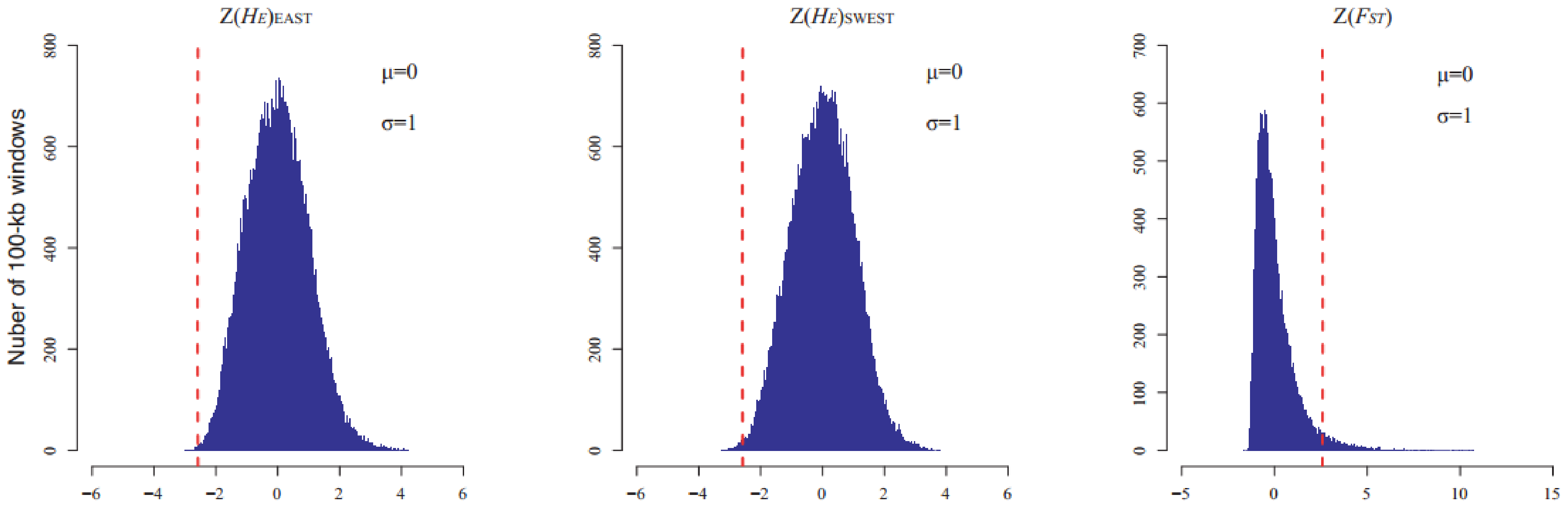

Supplementary Fig. 18. Distribution of heterozygosity calculated with 100 kb windows. Analyses were performed after Z-transformed in EAST (EAST_Z(H_E)) and SWEST (SWEST_Z(H_E)), respectively, as well as Z(F_{ST}) between EAST and SWEST. The dashed vertical red lines represent the thresholds at 99% confidence interval (μ, average; σ, standard deviation). Source data are provided in a Source Data file.

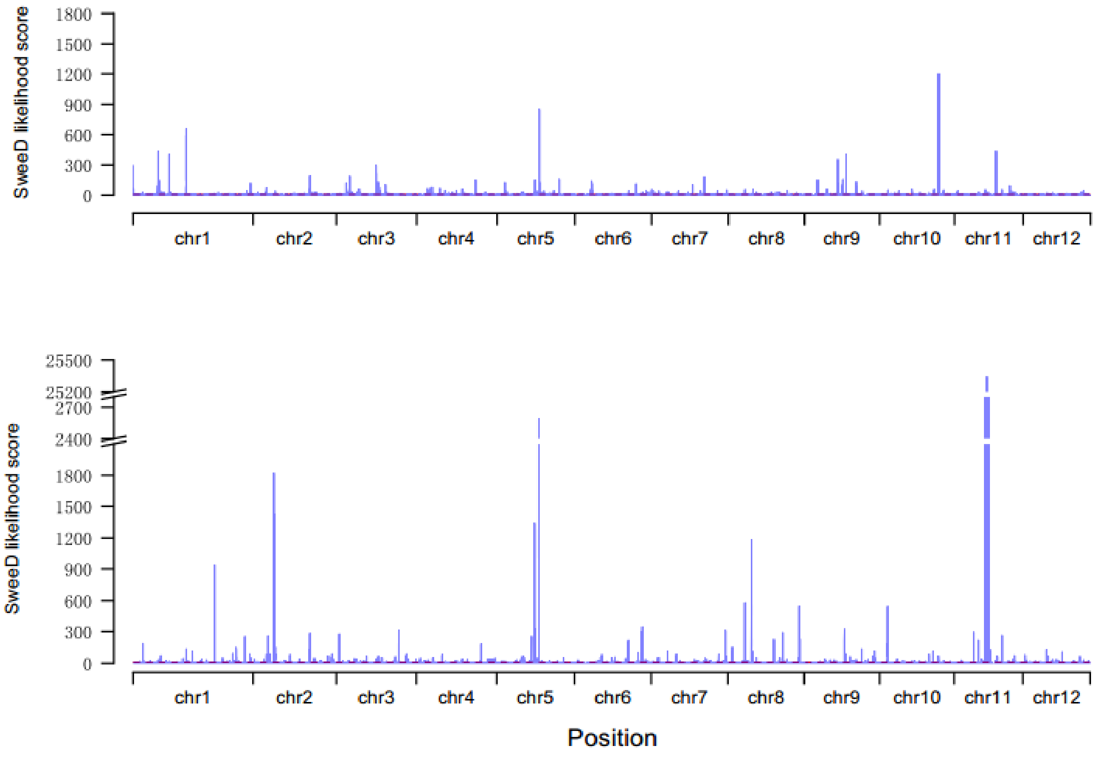

Supplementary Fig. 19. Selective sweep analysis in the EAST and SWEST groups by SweeD. Distribution of the regions under selection in EAST-g (**a**) and SWEST-g (**b**) along 12 chromosomes, respectively. A total of 903 regions were identified in both EAST-g and SWEST-g.

Supplementary Table 1. Information on the ginkgo samples collected outside China.

No.	Population code	Country	State/Province	No. trees sampled
1	KOR	South Korea	Gyeongsangbuk-do	6
2			Incheon	2
3			Jeollabuk-do	2
4			Chungcheongna-do	7
5			Gyeongsangna-do	1
6			Jeollana-do	2
7			Gangwon-do	1
8	KYS	Japan	Fukuoka	5
9			Kumamoto	1
10			Kagoshima	1
11			Oita	3
12			Saga	2
13	HNS	Japan	Tokyo	3
14			Ibaraki	1
15			Tochigi	2
16			Chiba	4
17			Yamaguchi	1
18			Niigata	1
19			Fukui	2
20			Yamanashi	1
21			Shizuoka	1
22			Aomori	1
23	SKK	Japan	Tokushima	1
24			Kochi	1
25			Ehime	3
26	EUR	Austria	Vienna	4
27		France	Générargues	3
28			Paris	1
29		Germany	Baden-Württemberg	1
30			Thüringen	4
31		Netherlands	Gelderland	2
32		United Kingdom	London	1
33	USA	USA	Pennsylvania	2
34			Massachusetts	3
35			New York	1
36			Illinois	2
37			South Carolina	1
38			Alabama	1
39			North Carolina	4
40			Wisconsin	1
41			Idaho	1

Supplementary Table 2. Comparison of SNP consistency between Illumina and BGISEQ-500 sequencing platforms by resequencing 14 individuals using both platforms.

Filtering condition	# detected SNPs	# filtered SNPs	#SNPs at different confidence levels	# overlap SNPs	Percent of overlap SNPs
No filter	84,359,357	-	84,359,357	83,179,179	98.60%
quality>100; depth>5	84,359,357	20,091,716	64,267,641	63,971,192	99.54%
quality>200; depth>10	84,359,357	33,975,593	50,383,764	50,173,727	99.58%
quality>300; depth>15	84,359,357	43,657,544	40,701,813	40,536,135	99.59%
quality>400; depth>20	84,359,357	50,620,231	33,739,126	33,604,832	99.60%
quality>500; depth>25	84,359,357	56,045,750	28,313,607	28,204,124	99.61%

Supplementary Table 3. Pairwise F_{ST} values between lineages and number of identical sequences within and between lineages.

	EAST	SOUTH	NORTH	SWEST
EAST	78	0.108	0.101	0.162
SOUTH	9	86	0.111	0.206
NORTH	15	30	124	0.036
SWEST	20	15	38	78

Note: Pairwise F_{ST} was calculated based on whole chloroplast genome sequences. Above and below the diagonal: pairwise F_{ST} and number of exactly identical sequences, respectively.

Supplementary Table 4. Genetic diversity of four lineages of ginkgo trees calculated based on whole chloroplast genome sequences.

**Lineage**	***N***	***S***	***H***	**π (10^{-4})**
EAST	92	63	7	0.96
SOUTH	91	63	8	0.44
NORTH	148	98	11	1.40
SWEST	115	68	7	1.70
**Total**	446	128	22	1.31

Note: *N*, trees sampled; *S*, number of segregating sites; *H*, number of haplotypes; π, nucleotide diversity.

Supplementary Table 5. Comparison of demographic models analyzed with *fastsimcoal2* for the four ginkgo lineages.

Model	Ancestor*	Max (log 10 (Est_hood)	No. estimated parameters	AIC*	ΔAIC	AIC's weight (w)
Model1	Putative	-5806	8	11628	28	~0
Model2	Putative	-6037	14	12102	502	~0
Model3	Putative	-5791	9	11600	0	~1
Model4	Putative	-5912	15	11854	254	~0
Model5	Putative	-5800	9	11618	18	~0
Model6	Putative	-5905	15	11840	240	~0
Model7	Putative	-5814	8	11644	44	~0
Model8	Putative	-6120	14	12268	668	~0
Model9	Putative	-5813	9	11644	44	~0
Model10	Putative	-6074	15	12178	578	~0
Model1a	EAST	-5831	8	11678	66	~0
Model2a	EAST	-5921	14	11870	258	~0
Model3a	EAST	-5797	9	11612	0	~1
Model4a	EAST	-6099	15	12228	616	~0
Model5a	EAST	NA	NA	NA	NA	NA
Model6a	EAST	NA	NA	NA	NA	NA
Model7a	EAST	-5805	8	11626	14	~0
Model8a	EAST	-6109	14	12246	634	~0
Model9a	EAST	-5803	9	11624	12	~0
Model10a	EAST	-6074	15	12178	566	~0
Model1b	SWEST	-5823	8	11662	58	~0
Model2b	SWEST	-6139	14	12306	702	~0
Model3b	SWEST	-5793	9	11604	0	~1
Model4b	SWEST	-6122	15	12274	670	~0
Model5b	SWEST	-5812	9	11642	38	~0
Model6b	SWEST	-6111	15	12252	648	~0
Model7b	SWEST	-5806	8	11628	24	~0
Model8b	SWEST	-6085	14	12198	594	~0
Model9b	SWEST	-5807	9	11632	28	~0
Model10b	SWEST	-6074	15	12178	574	~0

Note: Three possibilities were considered for the ancestor: 1) Putative, the four groups derive from a putative ancestor; 2) EAST, the EAST lineage is assumed to be the ancestor; 3) SWEST, the SWEST lineage is assumed to be the ancestor. NA indicates the absence of such a model. AIC, Akaike's information criterion value; ΔAIC, difference in AIC value from that of the strongest model; w, AIC weight

Supplementary Table 6. Inferred demographic parameters of the best-fitting demographic model shown in Supplementary Fig. 17.

Parameter	Point estimation	95% CI lower bound	95% CI upper bound
Ne_{ANC}	52,750	17,438	86,493
Ne_{EAST}	31,784	30,628	32,681
Ne_{SOUTH}	24,829	22,467	26,741
Ne_{NORTH}	29,062	16,361	29,096
Ne_{SWEST}	32,093	28,912	32,592
T_1	515,780	489,340	613,980
T_2	318,120	294,180	329,080
T_3	139,260	82,160	141,180
R1	0.7155	0.6421	0.7121

Supplementary Table 7. Relative importance of 19 bioclimatic variables in the distribution of ginkgo trees.

Variable	Interpretation	Percentage contribution				Permutation importance			
		China		EAST	SWEST	China		EAST	SWEST
		19bios	7bios			19bios	7bios		
BIO1	Annual mean temperature	7.7		0	0	17.4	-	0	0.1
**BIO2**	**Mean diurnal range**	3.3	4.4	2.5	4.5	2.9	5.2	0	0.3
BIO3	Isothermality (BIO2/BIO7)	2	-	1.9	0.3	2.2	-	26.1	0
**BIO4**	**Temperature seasonality**	4.2	10.7	25.4	16.6	3.4	3.3	0.7	0
BIO5	Max temperature of warmest month	2.4	-	0	0	3.4	-	0	0
BIO6	Min temperature of coldest month	6.3	-	3.3	1.9	4.3	-	43.3	10.8
BIO7	Temperature annual range (BIO5-BIO6)	1.7	-	0	0	8	-	0	0.1
**BIO8**	**Mean temperature of wettest quarter**	4.2	8.8	5.3	1.1	10.5	3.6	5.2	0
BIO9	Mean temperature of driest quarter	1.4	-	0.2	1.1	0.8	-	0	74
BIO10	Mean temperature of warmest quarter	2.2		0.9	0	4.7	-	0	0
**BIO11**	**Mean temperature of coldest quarter**	33.4	63.7	2.8	0	4.2	72.8	22.8	0
BIO12	Annual precipitation	3	-	0	0.6	5.2	-	0	6.9
BIO13	Precipitation of wettest month	2.3	-	0	0	3.2	-	0	0
**BIO14**	**Precipitation of driest month**	12.8	1.2	0	8.3	4.8	3.1	0	0.9
**BIO15**	**Precipitation seasonality**	5.8	6.2	0	3.9	1.7	6	0	0
BIO16	Precipitation of wettest quarter	1.2	-	0	3.9	5.1	-	0	0
BIO17	Precipitation of driest quarter	1	-	0	1.3	4.4	-	0	6.7
**BIO18**	**Precipitation of warmest quarter**	3.2	4.9	57.4	47.9	8.5	6	1.8	0.1
BIO19	Precipitation of coldest quarter	2	-	0.2	8.8	5.1	-	0	0

Note: The variables in bold represent variables with the most important contribution (significantly higher value than the other variables)

Supplementary Table 8. Information on the EAST and SWEST groups used for analyses of environmental adaptation and natural selection.

Group	Sample size	No. SNPs in dataset0 number	SNP number after filtering	Filtering ratio	H_E	F_{ST}
EAST-g	58	161,040,296	112,233,015	30.31%	0.2154	-
SWEST-g	58	161,040,296	101,447,217	37.01%	0.2402	-
Total	116	161,040,296	129,271,535	19.73%	0.1988	0.0637

Supplementary Table 9. GO enrichment of the EAST and SWEST groups.

Group	GO_ID	GO_Term	*P*-value†
EAST-g	GO:0006869	lipid transport	6.53E-06
	GO:0015914	phospholipid transport	0.001264
	GO:0033036	macromolecule localization	0.001937
	GO:0006665	sphingolipid metabolic process	0.003798
	GO:0071702	organic substance transport	0.005049
	GO:0006122	mitochondrial electron transport, ubiquinol to cytochrome C	0.013808
	GO:0045747	positive regulation of notch signaling pathway	0.013808
	GO:0009067	aspartate family amino acid biosynthetic process	0.020010
	**GO:0006950**	**response to stress**	**0.023346**
	**GO:0006952**	**defense response**	**0.024541**
	GO:0006810	transport	0.026964
	GO:0006562	proline catabolic process	0.027427
	GO:0042254	ribosome biogenesis	0.029901
	GO:0007049	cell cycle	0.049427
SWEST-g	**GO:0051716**	**cellular response to stimulus**	**0.005259**
	GO:0006000	fructose metabolic process	0.012581
	GO:0042450	arginine biosynthetic process via ornithine	0.012581
	GO:1901068	guanosine-containing compound metabolic process	0.023345
	GO:0007154	cell communication	0.023780
	GO:0006434	seryl-tRNA aminoacylation	0.025004
	**GO:0010038**	**response to metal ion**	**0.025004**
	GO:0007165	signal transduction	0.026321
	GO:0007155	cell adhesion	0.037273
	GO:0000724	double-strand break repair via homologous recombination	0.037273
	GO:0015940	pantothenate biosynthetic process	0.049388
	GO:0006003	fructose 2,6-bisphosphate metabolic process	0.049388
	GO:0006625	protein targeting to peroxisome	0.049388
	GO:0006165	nucleoside diphosphate phosphorylation	0.049388
	GO:0006183	GTP biosynthetic process	0.049388
	GO:0006228	UTP biosynthetic process	0.049388
	GO:0006241	CTP biosynthetic process	0.049388

Note: Terms in bold face are those involving responses to biotic and abiotic factors. † *P*-values were calculated by Fisher's exact test.

Supplementary Table 10. The genes that were identified by two approaches.

Group	Gene	Chromosome	H_E	$Z(H_E)$	F_{ST}	$Z(F_{ST})$	SweeD likelihood
	Gb_18600	10	0.0656	-2.6216	0.2469	3.7771	22.98017
	Gb_18601	10	0.0652	-2.6287	0.2591	4.0281	14.48341
EAST-g	Gb_18614	10	0.0661	-2.6136	0.2548	3.9405	22.98017
	**Gb_18615**	**10**	**0.0628**	**-2.6704**	**0.2381**	**3.595**	**14.48341**
	Gb_18616	10	0.0610	-2.7023	0.1936	2.6784	12.93183
	Gb_04748	11	0.0774	-2.7289	0.1909	2.6225	9260.24300
	Gb_05347	11	0.0841	-2.6172	0.2115	3.0463	6258.25900
	Gb_05350	11	0.0771	-2.7343	0.2151	3.122	7881.48800
	Gb_05351	11	0.0776	-2.7264	0.2260	3.3449	7925.62900
	Gb_05352	11	0.0776	-2.7264	0.2260	3.3449	9976.74100
	**Gb_11158**	**11**	**0.0836**	**-2.6253**	**0.2335**	**3.5003**	**2768.59000**
	Gb_12482	11	0.0851	-2.6013	0.2371	3.5742	3002.80700
	Gb_12754	11	0.0760	-2.7525	0.2216	3.2546	12988.70000
	Gb_12756	11	0.0800	-2.6857	0.2078	2.9705	7434.91500
	Gb_13752	2	0.0793	-2.6983	0.2531	3.9045	34.07885
SWEST-g	Gb_13753	2	0.0524	-3.1485	0.3005	4.8826	22.03867
	**Gb_26523**	**11**	**0.0853**	**-2.5976**	**0.2259**	**3.3437**	**12.70914**
	Gb_26524	11	0.0792	-2.6991	0.1912	2.6291	46.07054
	Gb_26528	11	0.0765	-2.744	0.2287	3.4007	5239.82800
	Gb_28299	11	0.0830	-2.6368	0.1957	2.7206	357.98420
	**Gb_36252**	**11**	**0.0696**	**-2.8608**	**0.2199**	**3.2201**	**10559.63000**
	Gb_38267	11	0.0768	-2.7405	0.1994	2.7971	1675.81700
	Gb_38268	11	0.0739	-2.7882	0.1949	2.7058	3526.44700
	Gb_38270	11	0.0794	-2.6968	0.2048	2.9083	3911.26000
	Gb_38271	11	0.0785	-2.7112	0.2221	3.2646	2217.56600

Note: Genes in bold face are those involving adaptation of ginkgo to local environments.

Supplementary Table 11. Eight high-depth sequenced trees used to infer demographic history using PSMC.

Sample	Lineage	Population code	Locality
TM106	EAST	TM	Mt. Tianmu, Zhejiang, China
TM169	EAST	TM	Mt. Tianmu, Zhejiang, China
JFDL33	SWEST	JF	Mt. Jinfo, Chongqing, China
JFDL39	SWEST	JF	Mt. Jinfo, Chongqing, China
GXXA02	SOUTH	GXXA	Xingan, Guangxi, China
ESY20	NORTH	ES	Enshi, Hubei, China
ESY6	NORTH	ES	Enshi, Hubei, China
HESX1	NORTH	HESX	Songxian, Henan, China

Note: Sequencing depths of eight high-depth sequenced individuals were 30.2X (TM106), 30.6X (TM169), 29.6X (JFDL33), 27.9X (JFDL39), 28.4X (GXXA02), 28.3 (ESY20), 29.8X (ESY6) and 29.2X (HESX1), with an average of 29X.

Supplementary References

1. Hori, T. *et al.* in *Ginkgo Biloba A Global Treasure - From Biology to Medicine* (Springer, 1997).
2. Werth, A. J. & Shear, W. A. The evolutionary truth about living fossils. *American Scientist* **102**, 434 (2014).
3. Ran, J. H., Shen, T. T., Wang, M. M. & Wang, X. Q. Phylogenomics resolves the deep phylogeny of seed plants and indicates partial convergent or homoplastic evolution between Gnetales and angiosperms. *Proc. R. Soc. B* **285**, 20181012 (2018).
4. Wu, C. S., Chaw, S. M. & Huang, Y. Y. Chloroplast phylogenomics indicates that *Ginkgo biloba* is sister to cycads. *Genome Biology and Evolution* **5**, 243-254 (2013).
5. Uemura, K. in *Ginkgo Biloba A Global Treasure.* (Springer, 1997).
6. Zhou, Z. Y. An overview of fossil Ginkgoales. *Palaeoworld* **18**, 1-22 (2009).
7. Crane, P. *Ginkgo:The Tree That Time Forgot*. (Yale University Press, 2013).
8. Del, T. P. Ginkgos and people - a thousand years of interaction. *Arnoldia* **51**, 2-15 (1991).
9. Gong, W., Chen, C., Dobeš, C., Fu, C. X. & Koch, M. A. Phylogeography of a living fossil: Pleistocene glaciations forced *Ginkgo biloba* L.(Ginkgoaceae) into two refuge areas in China with limited subsequent postglacial expansion. *Molecular Phylogenetics and Evolution* **48**, 1094-1105 (2008).
10. Zhao, Y. P., Paule, J., Fu, C. X. & Koch, M. A. Out of China: Distribution history of *Ginkgo biloba* L. *Taxon* **59**, 495-504 (2010).
11. Major, R. T. The ginkgo, the most ancient living tree: The resistance of *Ginkgo biloba* L. to pests accounts in part for the longevity of this species. *Science* **157**, 1270-1273 (1967).
12. Guan, R. *et al.* Draft genome of the living fossil *Ginkgo biloba*. *Gigascience* **5**, 49 (2016).
13. Hohmann, N. *et al. Ginkgo biloba*'s footprint of dynamic Pleistocene history dates back only 390,000 years ago. *BMC Genomics* **19**, 299 (2018).
14. Shen, L. *et al.* Genetic variation of *Ginkgo biloba* L. (Ginkgoaceae) based on cpDNA PCR-RFLPs: Inference of glacial refugia. *Heredity* **94**, 396-401 (2005).
15. Zhao, Y. P. *et al.* Incongruent range dynamics between co-occurring Asian temperate tree species facilitated by life history traits. *Ecology and Evolution* **6**, 2346-2358 (2016).
16. Tang, C. Q. *et al.* Evidence for the persistence of wild *Ginkgo biloba* (Ginkgoaceae) populations in the Dalou Mountains, southwestern China. *American Journal of Botany* **99**, 1408-1414 (2012).
17. Murray, M. G. & Thompson, W. F. Rapid isolation of high molecular weight plant DNA. *Nucleic Acids Research* **8**, 4321-4325 (1980).
18. Huang, J. *et al.* A reference human genome dataset of the BGISEQ-500 sequencer. *Gigascience* **6**, 1-9 (2017).

19. Chen, Y. *et al.* SOAPnuke: A MapReduce acceleration-supported software for integrated quality control and preprocessing of high-throughput sequencing data. *Gigascience* **7**, 1-6, doi:10.1093/gigascience/gix120 (2018).
20. Depristo, M. A. *et al.* A framework for variation discovery and genotyping using next-generation DNA sequencing data. *Nature Genetics* **43**, 491-498 (2011).
21. Alexander, D. H., Novembre, J. & Lange, K. Fast model-based estimation of ancestry in unrelated individuals. *Genome Research* (2009).
22. Chang, C. C. *et al.* Second-generation PLINK: Rising to the challenge of larger and richer datasets. *Gigascience* **4**, 7 (2015).
23. Purcell, S. *et al.* PLINK: A tool set for whole-genome association and population-based linkage analyses. *American Journal of Human Genetics* **81**, 559-575, doi:10.1086/519795 (2007).
24. Tamura, K., Dudley, J., Nei, M. & Kumar, S. MEGA4: Molecular evolutionary genetics analysis (MEGA) software version 4.0. *Molecular Biology and Evolution* **24**, 1596-1599 (2007).
25. Nystedt, B. *et al.* The Norway spruce genome sequence and conifer genome evolution. *Nature* **497**, 579-584 (2013).
26. Wan, T. *et al.* A genome for gnetophytes and early evolution of seed plants. *Nature Plants* **4**, 82 (2018).
27. Project, A. G. The Amborella genome and the evolution of flowering plants. *Science* **342**, 1467 (2013).
28. Tuskan, G. A. *et al.* The genome of black cottonwood, *Populus trichocarpa* (Torr. & Gray). *Science* **313**, 1596-1604 (2006).
29. Grabherr, M. G. *et al.* Trinity: Reconstructing a full-length transcriptome without a genome from RNA-Seq data. *Nature Biotechnology* **29**, 644 (2011).
30. Pertea, G. *et al.* TIGR Gene Indices clustering tools (TGICL): a software system for fast clustering of large EST datasets. *Bioinformatics* **19**, 651-652 (2003).
31. Yang, Z. PAML 4: Phylogenetic analysis by maximum likelihood. *Molecular Biology and Evolution* **24**, 1586-1591 (2007).
32. Buschiazzo, E., Ritland, C., Bohlmann, J. & Ritland, K. Slow but not low: Genomic comparisons reveal slower evolutionary rate and higher dN/dS in conifers compared to angiosperms. *BMC Evolutionary Biology* **12**, 8 (2012).
33. Schiffels, S. & Durbin, R. Inferring human population size and separation history from multiple genome sequences. *Nature Genetics* **46**, 919-925 (2014).
34. Excoffier, L., Dupanloup, I., Huertasánchez, E., Sousa, V. C. & Foll, M. Robust demographic inference from genomic and SNP data. *PLoS Genetics* **9**, e1003905 (2013).
35. Gutenkunst, R. N., Hernandez, R. D., Williamson, S. H. & Bustamante, C. D. Inferring the joint demographic history of multiple populations from multidimensional SNP frequency data. *PLoS Genetics* **5**, e1000695 (2009).

36. Jing, W., Street, N. R., Scofield, D. G. & Ingvarsson, P. K. Variation in linked selection and recombination drive genomic divergence during allopatric speciation of European and American aspens. *Molecular Biology and Evolution* **33**, 1754-1767 (2016).
37. Lanier, H. C., Massatti, R., He, Q., Olson, L. E. & Knowles, L. L. Colonization from divergent ancestors: Glaciation signatures on contemporary patterns of genomic variation in collared pikas (*Ochotona collaris*). *Molecular Ecology* **24**, 3688-3705 (2015).
38. Kumar, S., Stecher, G., Suleski, M. & Hedges, S. B. TimeTree: A resource for timelines, timetrees, and divergence times. *Molecular Biology and Evolution* **34**, 1812-1819 (2017).
39. Hill, K. Diversity and evolution of gymnosperms. *Plant diversity and evolution: Genotypic and phenotypic variation in higher plants/RJ Henry–CABI Publishing* **25**, 45 (2005).
40. Peterson, B. J. & Graves, W. R. Chloroplast phylogeography of *Dirca palustris* L. indicates populations near the glacial boundary at the Last Glacial Maximum in eastern North America. *Journal of Biogeography* **43**, 314-327 (2016).
41. Call, A. *et al.* Genetic structure and post- glacial expansion of *Cornus florida* L. (Cornaceae): Integrative evidence from phylogeography, population demographic history, and species distribution modeling. *Journal of Systematics and Evolution* **54**, 136-151 (2016).
42. Phillips, S. J., Anderson, R. P. & Schapire, R. E. Maximum entropy modeling of species geographic distributions. *Ecological Modelling* **190**, 231-259 (2006).
43. Phillips, S. J. & Dudík, M. Modeling of species distributions with Maxent: New extensions and a comprehensive evaluation. *Ecography* **31**, 161-175 (2008).
44. Xing, S. *Ginkgo Germplasm Resources in China.* (China Forestry Publishing House, 2013).
45. Hijmans, R. J., Cameron, S. E., Parra, J. L., Jones, P. G. & Jarvis, A. Very high resolution interpolated climate surfaces for global land areas. *International Journal of Climatology* **25**, 1965-1978 (2010).
46. Ottobliesner, B. L., Marshall, S. J., Overpeck, J. T., Miller, G. H. & Hu, A. Simulating Arctic climate warmth and icefield retreat in the last interglaciation. *Science* **311**, 1751-1753 (2006).
47. Fawcett, T. An introduction to ROC analysis. *Pattern Recognition Letters* **27**, 861-874 (2006).
48. Axelsson, E. *et al.* The genomic signature of dog domestication reveals adaptation to a starch-rich diet. *Nature* **495**, 360 (2013).
49. Mace, E. S. *et al.* Whole-genome sequencing reveals untapped genetic potential in Africa's indigenous cereal crop sorghum. *Nature Communications* **4**, 2320 (2013).
50. Vitti, J. J., Grossman, S. R. & Sabeti, P. C. Detecting natural selection in genomic data. *Annual Review of Genetics* **47**, 97-120 (2013).
51. Rubin, C. J. *et al.* Whole-genome resequencing reveals loci under selection during chicken domestication. *Nature* **464**, 587 (2010).
52. Weir, B. S. & Cockerham, C. C. Estimating F-statistics for the analysis of population structure. *Evolution* **38**, 1358-1370 (1984).

53. Danecek, P. *et al.* The variant call format and VCFtools. *Bioinformatics* **27**, 2156-2158 (2011).

54. Nielsen, R. *et al.* Genomic scans for selective sweeps using SNP data. *Genome Research* **15**, 1566 (2005).

55. Pavlidis, P., Živkovic, D., Stamatakis, A. & Alachiotis, N. SweeD: Likelihood-based detection of selective sweeps in thousands of genomes. *Molecular Biology and Evolution* **30**, 2224 (2013).

56. Alexa, A., Rahnenführer, J. & Lengauer, T. Improved scoring of functional groups from gene expression data by decorrelating GO graph structure. *Bioinformatics* **22**, 1600 (2006).

57. Hammond, G. R. & Balla, T. Polyphosphoinositide binding domains: Key to inositol lipid biology. *Biochimica Et Biophysica Acta (BBA)-Molecular and Cell Biology of Lipids* **1851**, 746-758 (2015).

58. Desai, M. *et al.* Two inositol hexakisphosphate kinases drive inositol pyrophosphate synthesis in plants. *Plant Journal* **80**, 642-653 (2014).

59. Laha, D. *et al.* VIH2 Regulates the synthesis of inositol pyrophosphate InsP8 and jasmonate-dependent defenses in Arabidopsis. *Plant Cell* **27**, 1082-1097 (2015).

60. Deyoung, B. J. & Innes, R. W. Plant NBS-LRR proteins in pathogen sensing and host defense. *Nature Immunology* **7**, 1243 (2006).

61. Marone, D., Russo, M., Laidò, G., Leonardis, A. D. & Mastrangelo, A. Plant nucleotide binding site–leucine-rich repeat (NBS-LRR) genes: Active guardians in host defense responses. *International Journal of Molecular Sciences* **14**, 7302-7326 (2013).

62. Mchale, L., Tan, X., Koehl, P. & Michelmore, R. W. Plant NBS-LRR proteins: Adaptable guards. *Genome Biology* **7**, 1-11 (2006).

63. Diévart, A. & Clark, S. E. Using mutant alleles to determine the structure and function of leucine-rich repeat receptor-like kinases. *Current Opinion in Plant Biology* **6**, 507-516 (2003).

64. Hong, S. W., Jon, J. H., Kwak, J. M. & Nam, H. G. Identification of a receptor-like protein kinase gene rapidly induced by abscisic acid, dehydration, high salt, and cold treatments in *Arabidopsis thaliana. Plant Physiology* **113**, 1203-1212 (1997).

65. Nodine, M. D. & Tax, F. E. Two receptor-like kinases required together for the establishment of Arabidopsis cotyledon primordia. *Developmental Biology* **314**, 161-170 (2008).

66. Osakabe, Y. *et al.* Leucine-rich repeat receptor-like kinase1 is a key membrane-bound regulator of abscisic acid early signaling in Arabidopsis. *Plant Cell* **17**, 1105-1119 (2005).

67 Lin, C.-P., Wu, C.-S., Huang, Y.-Y., Chaw, S.-M. The complete chloroplast genome of *Ginkgo biloba* reveals the mechanism of inverted repeat contraction. *Genome Biol Evol*. **4**, 374–381 (2012).

68 Jin, J.J., Yu W. B., Yang, J. B., Song, Y., Yi T. S., Li, D. Z. GetOrganelle: A simple and fast pipeline for de novo assembly of a complete circular chloroplast genome using genome skimming data. *bioRxiv*, 256479. http://doi.org/10.1101/256479 (2018).

69. Nakamura, T. , Yamada, K. D. , Tomii, K. , Katoh, K. , & Hancock, J. Parallelization of mafft for large-scale multiple sequence alignments. *Bioinformatics* **14**, 2490-2492 (2018).

70 Tillich, M., Lehwark, P., Pellizzer, T., Ulbricht-Jones, E.S., Fischer, A., Bock, R., Greiner ,S. GeSeq – versatile and accurate annotation of organelle genomes. *Nucleic Acids Research* **45**, W6-W11 (2017).

71 Leigh, J. W, & Bryant, D. PopART: Full-feature software for haplotype network construction. *Methods Ecol Evol* **6**, 1110–1116 (2015).

72 Pfeifer, B., Wittelsbürger, U., Ramos-Onsins, S. E., Lercher, M. J. PopGenome: An efficient Swiss Army Knife for population genomic analyses in *R. Molecular Biology and Evolution,* **31**, 1929-1936 (2014).

73 Fithian, W., Elith, J., Hastie, T. & Keith, D. A. Bias correction in species distribution models: pooling survey and collection data for multiple species. *Molecular Ecology and Evolution* **6**, 424-438 (2015).

74 Zhou, W. *et al.* Resolving relationships and phylogeographic history of the *Nyssa sylvatica* complex using data from RAD-seq and species distribution modeling. *Molecular Phylogenetics and Evolution* **126**,1-16 (2018).

75 Efron, B. Bootstrap methods: another look at the jackknife. In: *The Annals of Statistics*. Springer, pp. 1–26. doi: 10.1214/aos/1176344552 (1992).

76 Simpson, L., Clements, M.A., Crayn, D.M., Schulte, K. Evolution in Australia's mesic biome under past and future climates: insights from a phylogenetic study of the Australian Rock orchids (Dendrobium speciosum complex, Orchidaceae). *Mol. Phylogenet. Evol.* **118**, 32–46 (2017).

77 Graham, M.H. Confronting multicollinearity in ecological multiple regression. *Ecol. Lett.* **84**, 2809–2815 (2003).

78 Pearson, R.G., Raxworthy, C.J., Nakamura, M., Townsend Peterson, A. Predicting species distributions from small numbers of occurrence records: A test case using cryptic geckos in Madagascar. *J. Biogeogr*. **34**, 102–117 (2006).
