# Supplementary material for: Resequencing 545 ginkgo genomes across the world reveals the evolutionary history of the living fossil
Source: Nat Commun. 2019 Sep 13;10:4201. doi: 10.1038/s41467-019-12133-5 (PMC6744486; doi:10.1038/s41467-019-12133-5)
Supplement: Supplementary file 3 — Reporting Summary [file 41467_2019_12133_MOESM3_ESM.pdf]

nature research

Corresponding author(s): Song Ge

Last updated by author(s): 2019-8-13

Reporting Summary

Nature Research wishes to improve the reproducibility of the work that we publish. This form provides structure for consistency and transparency in reporting. For further information on Nature Research policies, see Authors & Referees and the Editorial Policy Checklist .

Statistics

For all statistical analyses, confirm that the following items are present in the figure legend, table legend, main text, or Methods section.

n/a	Confirmed	
☐	☒	The exact sample size (*n*) for each experimental group/condition, given as a discrete number and unit of measurement
☐	☒	A statement on whether measurements were taken from distinct samples or whether the same sample was measured repeatedly
☐	☒	The statistical test(s) used AND whether they are one- or two-sided<br>*Only common tests should be described solely by name; describe more complex techniques in the Methods section.*
☒	☐	A description of all covariates tested
☒	☐	A description of any assumptions or corrections, such as tests of normality and adjustment for multiple comparisons
☐	☒	A full description of the statistical parameters including central tendency (e.g. means) or other basic estimates (e.g. regression coefficient) AND variation (e.g. standard deviation) or associated estimates of uncertainty (e.g. confidence intervals)
☐	☒	For null hypothesis testing, the test statistic (e.g. *F*, *t*, *r*) with confidence intervals, effect sizes, degrees of freedom and *P* value noted<br>*Give P values as exact values whenever suitable.*
☒	☐	For Bayesian analysis, information on the choice of priors and Markov chain Monte Carlo settings
☒	☐	For hierarchical and complex designs, identification of the appropriate level for tests and full reporting of outcomes
☒	☐	Estimates of effect sizes (e.g. Cohen's *d*, Pearson's *r*), indicating how they were calculated

Our web collection on statistics for biologists contains articles on many of the points above.

Software and code

Policy information about availability of computer code

Data collection	NA
Data analysis	Softwares used in this study include SOAPnuke (ver. 1.5.4), DRAGEN (http://www.edicogenome.com/), GATK 4.0 Haplotype Variant Caller, ADMIXTURE (ver. 1.3.0), PLINK (ver. 1.90), MEGA (ver. 4.0), fastsimcoal2, easySFS.py (https://github.com/isaacovercast/easySFS), MAXENT (v3.3.1), SweeD (version 3.1),

For manuscripts utilizing custom algorithms or software that are central to the research but not yet described in published literature, software must be made available to editors/reviewers. We strongly encourage code deposition in a community repository (e.g. GitHub). See the Nature Research guidelines for submitting code & software for further information.

Data

Policy information about availability of data

All manuscripts must include a data availability statement. This statement should provide the following information, where applicable:

- Accession codes, unique identifiers, or web links for publicly available datasets
- A list of figures that have associated raw data
- A description of any restrictions on data availability

The resequencing reads of gingko in this study have been deposited in NCBI Sequence Read Archive (SRA) under bioproject accession PRJNA478810 [https://www.ncbi.nlm.nih.gov/bioproject/PRJNA478810].
The source data underlying Figs 1a-c, 3a–c, 4b and Supplementary Figs 2, 3b, 5a-b, 6, 11a-b, 12, 13a-b, and 17 are provided as a Source Data file.

Field-specific reporting

Please select the one below that is the best fit for your research. If you are not sure, read the appropriate sections before making your selection.

☐ Life sciences ☐ Behavioural & social sciences ☒ Ecological, evolutionary & environmental sciences

For a reference copy of the document with all sections, see nature.com/documents/nr-reporting-summary-flat.pdf

Ecological, evolutionary & environmental sciences study design

All studies must disclose on these points even when the disclosure is negative.

Study description	This study was designed to establish a most comprehensive and robust evolutionary history of Ginkgo biloba using genome resequencing data.
Research sample	A total of 545 old ginkgo trees were collected from 51 populations across the world. This is a collection of nearly two-decade efforts. We sampled these trees either with a diameter at breast height (DBH) larger than 50 cm, corresponding to a minimum age of ~120 years, or with observed reproductive organs. Background information on populations such as habitats and historical references was also collected.
Sampling strategy	To address evolutionary history and human-aided introduction out of China, we tried to collect evidenced and putative natural populations in China as well as old ginkgo trees in Japan, Korea, Europe and USA.
Data collection	Field data were collected by authors from Zhejiang University and their collaborators. Genetic data were generated by authors from BGI-Qingdao.
Timing and spatial scale	Genetic data were generate at BGI, China, from July 2016 to September 2017
Data exclusions	No data were excluded.
Reproducibility	We called the SNPs using the same pipeline and parameters and compared the two SNP sets of these 14 samples generated by Illumina Hiseq2000 sequencing platform and BGISEQ-500 separately. We found that more than 98.6% of SNPs were shared by the two platforms in different filtering standards, suggesting the high consistence between the two sequencing platforms .
Randomization	Samples were grouped according to previous phylogeograhic evidence as well as to geographic regions. More samples were selected for the evidence natural populations relative to the introduced ones.
Blinding	All samples were numbered and subject to sequencing by the staff who know no information about the source of samples.

Did the study involve field work? ☒ Yes ☐ No

Field work, collection and transport

Field conditions	Nature reserves and villages in China, botanical gardens, temples or shrines, etc.
Location	51 populations across the world. Please see detailed information in Supplementary Table 1.
Access and import/export	We apply for collection to the administrative bureaus of natural reserves in China as well as to the temples and shrines with assistance of colleagues.
Disturbance	NA

Reporting for specific materials, systems and methods

We require information from authors about some types of materials, experimental systems and methods used in many studies. Here, indicate whether each material, system or method listed is relevant to your study. If you are not sure if a list item applies to your research, read the appropriate section before selecting a response.

Materials & experimental systems

n/a	Involved in the study
☒	☐ Antibodies
☒	☐ Eukaryotic cell lines
☒	☐ Palaeontology
☒	☐ Animals and other organisms
☒	☐ Human research participants
☒	☐ Clinical data

Methods

n/a	Involved in the study
☒	☐ ChIP-seq
☒	☐ Flow cytometry
☒	☐ MRI-based neuroimaging
