# Supplementary material for: Resequencing 545 ginkgo genomes across the world reveals the evolutionary history of the living fossil
Source: Nat Commun. 2019 Sep 13;10:4201. doi: 10.1038/s41467-019-12133-5 (PMC6744486; doi:10.1038/s41467-019-12133-5)
Supplement: Supplementary file 4 — Description of Additional Supplementary Files [file 41467_2019_12133_MOESM4_ESM.doc]

**Description of Additional Supplementary Files**

File Name: Supplementary Data 1

Description: Detailed information on the ginkgo populations in the present study.

File Name: Supplementary Data 2

Description: The statistics of raw reads, clean reads, reads mapping and sequencing depth.

File Name: Supplementary Data 3

Description: Genes with selection signals of the EAST and SWEST groups identified by Z(*H*_E_) and Z(*F*_ST_) computed by 100kb windows

File Name: Supplementary Data 4

Description: Genes with selection signals of the EAST and SWEST groups identified by Z(*H*_E_) and Z(*F*_ST_) computed by 50kb windows

File Name: Supplementary Data 5

Description: Genes with selection signals of the EAST and SWEST groups identified by Z(*H*_E_) and Z(*F*_ST_) computed by 200kb windows

File Name: Supplementary Data 6

Description: Genes with selection signals of the EAST and SWEST groups identified by SweeD.

File Name: Supplementary Data 7

Description: The genes that were identified by at least one method.
