# Supplementary material for: Resequencing 545 ginkgo genomes across the world reveals the evolutionary history of the living fossil
Source: Nat Commun. 2019 Sep 13;10:4201. doi: 10.1038/s41467-019-12133-5 (PMC6744486; doi:10.1038/s41467-019-12133-5)
Supplement: Supplementary file 2 — Peer Review [file 41467_2019_12133_MOESM2_ESM.pdf]

Reviewers' comments:

Reviewer #1 (Remarks to the Author):

In this study, Zhao et al. resequenced 545 accessions of ginkgo trees collected across the world. They found population expansion rather than reduction since the last glaciation, contradicting the widespread assumption that ginkgo has remained largely unchanged as indicated by the fossil evidence. Using population structure analysis, the authors found human-mediated dispersal of ginkgo out of the refugia in China and further introductions to East Asia, Europe and America. By characterization of bioclimatic variables, they found environmental changes and natural selection played important roles in shaping the geographic distribution of ginkgo. The manuscript was well written, and it fits the scope of the journal and would be of interest to readers.
Below are some suggestions for further improvement of the manuscript.
1. The availability of the sequence data is a valuable resource for ginkgo research. Where are the sequence data deposited? How to access? Please add relevant information on the manuscript.
2. Due to morphological stasis, the living gymnosperm diversity has long been assumed to represent the last remnants of their formidable past and so-called 'living fossils'. Contradicting this widespread assumption, authors found population expansion rather than reduction since the last glaciation. Similar discoveries on gymnosperm diversification were published in New Phytologist and Science as listed below. The two important references should not be missed in the manuscript.

Reviewer #2 (Remarks to the Author):

This is an interesting manuscript about the sequencing of genomes from 545 ginkgo trees from around the world, including several major populations within China as well as trees from Europe and the United States. The manuscript includes much novel information that should be of general interest to readers. The writing is appropriate and the figures with the paper (and also with the supplementary information) are of high quality and easy to follow.

There are many abbreviations used here, some of which are commonly used (SNP) whereas others (SDM, LGM, MAR, SWEST) are less familiar. Although these abbreviations shorten the paper and aid the reader, they can also be a bit confusing. There is quite a bit of jargon used here too.

The authors present a convincing case about the phylogeny and global population structure of ginkgos. I think they do an especially good job relating this to glacial refugia and adaptation to various bioclimatic variables (mostly relating to temperature and precipitation).

The authors also discuss the ways that the ginkgo tree is remarkably resistant to both biotic and abiotic stresses, which undoubtedly has aided its survival not only over an exceptionally long period of time (hence its description as a "living fossil") but also over a huge geographic area, including urban settings. My main recommendation is that the authors should emphasize this point more broadly, including in their abstract. The huge genome of this species—with its expanded "evolutionary capacitance," despite little external morphological change—surely also has contributed to its survival over time.

In summary, the authors do a good job describing their very informative study, but they can probably put it into an even broader evolutionary context than they do here.

This manuscript includes a great deal of information and is mostly well written, although it could use a bit of English editing.

Reviewer #3 (Remarks to the Author):

The authors presented a study focusing on the evolutionary history of Ginkgo biloba and using a simple genome-resequencing approach largely relying on a previously published genome.

The authors presented a spatio-temporal framework for Ginkgo evolutionary history simce that last 300,000 years. It has to be noted here, that most of the general findings are not new and have been already published by the same authors and based on different and other datasets. Although whole-genome data are more robust and maybe even meaningful, I do not see here a major achievement compared to earlier studies. Of course, there are some new findings (e.g., more detailed refuge area structure: 3rd refugium in S China), but key questions about human-mediated influence on gene pool structure and composition, human-mediated selection on sex-ratios, age-depending differentiation, etc. are still not answered.

Although the auhtors claimed with the title "545 Ginkgo genomes" to be analysed, it is quite different what you find with the paper. There it is 51 "populations" (with some within population sampling, of course), but populations have never been properly defined afterwards. So it remains unclear what "population" means. Throughout the whole paper this is later mostly referred to STRUCTURE analysis or simple "joined analysis" (Supp. Fig 3.4)... and I am missing any detailed population-genetic analysis highlighting processes as indicated above.

The authors - as mentioned - confirmed various numbers, which is great: divergence time estimates from previous studies, general signature of secondary contact and admixture, multiple-times out of China (human-mediated), ...
Some of them need to discussed and analysed more extensively. Effective population sizes, for example, vary with the presented paper drastically among algorithms used (Fig. 2). Similary, their 2016 paper in EcoEvol show also slightly different numbers. How is this explained? Why isn´t it set into the context of ecological niche modelling and available distriution ranges? How is this linked to respective woodland vegetation. I assume that Ginkgo is not dominating in any type of woodland vegetation.

A final remark, which I strongly suggest to be considered: Type of raw data should allow to (1) extract complete and highly quality plastid genome information. This data can be used to complement directly past work of the same authors. And (2) the same should be done for mitochondrial DNA. It should be possible to extract at least the entire "gene space". These data might help to interprete dispersal and migration events, since you two additional genomes can be considered.

In summary, the authors provided a nice story that might have the potential (with their generated raw data) to have some impact on scientific thinking in the field - however, as it stands respective questions and hypotheses have not been raised up ! It is a largely descriptive study.

I do not see any major technical ot statiscal flaws, but this largely depends on reliability of the reference genome, which I cannot comment on.

Responses to three reviewers

Reviewer #1 (Remarks to the Author):

In this study, Zhao et al. resequenced 545 accessions of ginkgo trees collected across the world. They found population expansion rather than reduction since the last glaciation, contradicting the widespread assumption that ginkgo has remained largely unchanged as indicated by the fossil evidence. Using population structure analysis, the authors found human-mediated dispersal of ginkgo out of the refugia in China and further introductions to East Asia, Europe and America. By characterization of bioclimatic variables, they found environmental changes and natural selection played important roles in shaping the geographic distribution of ginkgo. The manuscript was well written, and it fits the scope of the journal and would be of interest to readers.

Below are some suggestions for further improvement of the manuscript.

1. The availability of the sequence data is a valuable resource for ginkgo research. Where are the sequence data deposited? How to access? Please add relevant information on the manuscript.

Response: The reviewer might miss the section "Data availability" in the end of the text, in which we noted "the resequencing data in this study have been deposited in public databases, including both NCBI SRA database under BioProject accession numbers: PRJNA478810 and CNGB Nucleotide Sequence Archive under accession number: CNP0000136." As a routine, the data will be released once the paper is accepted.

----

2. Due to morphological stasis, the living gymnosperm diversity has long been assumed to represent the last remnants of their formidable past and so-called 'living fossils'. Contradicting this widespread assumption, authors found population expansion rather than reduction since the last glaciation. Similar discoveries on gymnosperm diversification were published in New Phytologist and Science as listed below. The two important references should not be missed in the manuscript.

Response: We really appreciate this reviewer for recommending the two references that provided case studies involving the diversification of cycads and other extant gymnosperms. We also cited one new publication (Dorsey *et al*. 2018. *AJB*) that addressed a similar question. These studies supported the argument that ancient lineages or "living fossils" might have survived by making adaptive shifts and thus be successful lineages.

Reviewer #2 (Remarks to the Author):

This is an interesting manuscript about the sequencing of genomes from 545 ginkgo trees from around the world, including several major populations within China as well as trees from Europe and the United States. The manuscript includes much novel information that should be of general interest to readers. The writing is appropriate and the figures with the

paper (and also with the supplementary information) are of high quality and easy to follow.

There are many abbreviations used here, some of which are commonly used (SNP) whereas others (SDM, LGM, MAR, SWEST) are less familiar. Although these abbreviations shorten the paper and aid the reader, they can also be a bit confusing. There is quite a bit of jargon used here too.

Response: Thanks for the suggestions. We have added the full names of the abbreviations with necessary explanations when they first appear in the main text and the supplementary files. We also checked through the manuscript and have provided explanations for some jargons (terms) for clarity in the revision.

The authors present a convincing case about the phylogeny and global population structure of ginkgos. I think they do an especially good job relating this to glacial refugia and adaptation to various bioclimatic variables (mostly relating to temperature and precipitation). The authors also discuss the ways that the ginkgo tree is remarkably resistant to both biotic and abiotic stresses, which undoubtedly has aided its survival not only over an exceptionally long period of time (hence its description as a "living fossil") but also over a huge geographic area, including urban settings. My main recommendation is that the authors should emphasize this point more broadly, including in their abstract. The huge genome of this species—with its expanded "evolutionary capacitance," despite little external morphological change—surely also has contributed to its survival over time.

In summary, the authors do a good job describing their very informative study, but they can probably put it into an even broader evolutionary context than they do here.

Response: We fully agree with this reviewer that we should put our findings "into an even broader evolutionary context than they do here". Actually, the main points in this study involved several longstanding and important questions such as (1) why living fossils look unchanged despite numerous speculations, (2) whether living fossils are underway to extinction, and (3) what roles human has played in the survival and spread of living fossils, all of which have been discussed in the Discussion. As recommended by this reviewer, we have emphasized these points in the Abstract and discussed these issues more thoroughly in the revised Discussion. Overall, we strengthened and highlighted the broad significance arising from this study in the different sections in the revised manuscript.

This manuscript includes a great deal of information and is mostly well written, although it could use a bit of English editing.

Response: In response to this point, we have sent out our revised manuscript to the American Journal Experts (AJE) to have made professional scientific editing (the editorial certificate is attached).

Reviewer #3 (Remarks to the Author):

The authors presented a study focusing on the evolutionary history of Ginkgo biloba and using a simple genome-resequencing approach largely relying on a previously published genome.

The authors presented a spatio-temporal framework for Ginkgo evolutionary history simce that last 300,000 years. It has to be noted here, that most of the general findings are not new and have been already published by the same authors and based on different and other datasets. Although whole-genome data are more robust and maybe even meaningful, I do not see here a major achievement compared to earlier studies. Of course, there are some new findings (e.g., more detailed refuge area structure: 3rd refugium in S China), but key questions about human-mediated influence on gene pool structure and composition, human-mediated selection on sex-ratios, age-depending differentiation, etc. are still not answered.

Response: We understand the concerns of this reviewer, which are relevant to the novelty and significance of this study. Here, we would provide our arguments for the novelty and achievements of this study as follows.

1) Indeed, several authors of the present paper have been working on this species for two decades and have published a series of papers focusing on population genetics, phylogeography and evolutionary history. As we indicated in the Introduction, however, several important questions remain unsolved mainly because all previous studies suffered from limited molecular makers used and the sampled populations/individuals were insufficient to cover the entire distribution range of ginkgo trees. Here we would highlight a few of points that we believe to be novel and of significance.

(i) In addition to confirming two refugia in the eastern China and southwestern China that were reported previously, we identified, for the first time, a new refugium in southern China in which all four ancient genetic components were found, and demonstrated that the populations in northern China (i.e., NORTH) were an admixed lineage that might form through expansions of the populations persisting in the refugia during the last interglacial (LIG), supporting the glacial admixture hypothesis.

(ii) Using the largest genome dataset so far, we elucidated the population dynamics of ginkgo populations in great details in terms of time and scale, indicating clearly that ginkgo populations underwent multiple cycles of expansion and reduction along with admixture of relict populations during the Pleistocene glaciations. These issues have been unsolved or debated for a long time.

(iii) We characterized the major bioclimatic variables that shaped the geographic distribution of ginkgo, and addressed the potential roles of natural selection in the survival and resilience of ginkgo populations based on genome scanning. These investigations laid an important basis for further studies of the potential mechanism of ginkgo’s survival and resilience.

(iv) We presented a scenario how ginkgo trees dispersed out of refugia and out of China based on multiple evidence/analyses, which refuted previous speculations on the human-mediated introduction history of ginkgo from Japan to Europe and America.

Together, taking advantages of the whole-genome sequencing data and sophisticated approaches, this study provides additional insights into the evolutionary history of this living fossil, which lays an important foundation for further studies addressing various questions involving living fossils using ginkgo as a model.

2) This reviewer raised an excellent point regarding in-depth investigations on additional key questions such as anthropogenic (human-mediated) influences on gene pool structure and composition, human-mediated selection on sex-ratios, age-depending differentiation, etc. We do agree that all these questions are important and worthwhile studying from different angles. Also, we believe that clear elucidations of population structure and evolutionary history are the basis for further in-depth investigations that could be unlikely addressed in a single study.

3) In response to these concerns, also as suggested by reviewer 2, we have added several sentences in the Abstract and the last paragraph of Introduction to emphasize the significance of this study in a broader evolutionary context.

Although the auhtors claimed with the title "545 Ginkgo genomes" to be analysed, it is quite different what you find with the paper. There it is 51 "populations" (with some within population sampling, of course), but populations have never been properly defined afterwards. So it remains unclear what "population" means. Throughout the whole paper this is later mostly referred to STRUCTURE analysis or simple "joined analysis" (Supp. Fig 3.4)... and I am missing any detailed population-genetic analysis highlighting processes as indicated above.

Response: We apologize that we have not made it clearer about the definition of "populations" although we tried to present them in Extended DATA Table 1. Probably we need describe our sampling strategy in more details. First, for simplicity, we would cite a textbook definition (Hartl and Clark. 2007. Principles of Population Genetics. p.45): "The word population has so far been used in an informal, intuitive sense to refer to a group of organisms belonging to the same species… living within a sufficiently restricted geographical area. …Such a population is often referred to as local population". The use of "population" in our study followed exactly the same definition, i.e., a group of individuals living within a restricted geographical area (of course, the same species). Under such a definition or concept, we collected 51 local populations with sample sizes ranging from one to 58 (see the Extended DATA Table 1), representing the entire gene pool of ginkgo around the world. Because of various sizes of local populations in our case, we merged them into groups or lineages (say, EAST, SWEST,…) before population genetic analysis was performed to avoid the impact of various sizes of different populations on the analyses. Such a sampling strategy and analytical practice have been

widely used in studies of natural populations, including humans (Skoglund *et al*. 2017. *Cell*; Mathieson *et al*. 2018. *Nature*), model species (Good *et al*. 2017. *Nature*; Murray *et al*. 2017. *Science*; Bay *et al*. 2018. *Science*), crops (Salojarvi *et al*. 2017. *Nat Genet*; Wu G *et al*. 2018. *Nature*), to name a few. The unbalanced population sizes were common to many rare or endangered species like living fossils. This is also the reason that we did not perform in-depth population genetics analyses as indicated by this reviewer in above point.

Despite these clarifications on "population", we realized that the terms "lineage, group, population, and refugia" were crossly used in the present manuscript, which led to unnecessary confusions. Therefore, in addition to clarifying the term "population", we used consistently these terms through the text as follows:

(i) lineage: a group of populations with similar genetic components as revealed by genetic analyses (i.e., NJ tree, STRUCTURE, PCA). We detected four lineages (i.e., EAST, SOUTH, NORTH, and SWEST) and deleted "group" in such cases to avoid confusion.

(ii) group: we used this term in one case only, i.e., "Analysis of footprints of natural selection". In this analysis, we sampled two groups of individuals (each consisting of 58 individuals) to represent the EAST and SWEST lineages (named as EAST-g and SWEST-g, respectively). Such a practice avoids the double meanings of "EAST" and "SWEST" in the original manuscript.

(iii) refugium (pl. refugia): used exclusively to refer to the area or place where the relict populations were found. Thus, we mentioned the three refugia as eastern China, southern China, and southwest China rather than EAST, SOUTH, SWEST.

Finally, we added and revised sentences to clarify these terms in relevant places across the text and Supplementary Information.

The authors - as mentioned - confirmed various numbers, which is great: divergence time estimates from previous studies, general signature of secondary contact and admixture, multiple-times out of China (human-mediated), ... Some of them need to discussed and analysed more extensively. Effective population sizes, for example, vary with the presented paper drastically among algorithms used (Fig. 2). Similary, their 2016 paper in EcoEvol show also slightly different numbers. How is this explained? Why isn´t it set into the context of ecological niche modelling and available distribution ranges? How is this linked to respective woodland vegetation. I assume that Ginkgo is not dominating in any type of woodland vegetation.

Response: We highly appreciate these constructive suggestions, which are really helpful in improving the quality of this work. Thus, we have made substantial revisions as follows.

1) First, we re-analyzed the demographic history using *fastsimcoal2* by adding new models (see the revised Supplementary Fig. 4.3). Importantly, based on the updated

analysis with 30 models derived from ten basic ones, we obtained several results that were congruent between PSMC and *fastsimcoal2* analyses and reasonable in terms of various population genetic parameters (see the revised Fig. 2). Specifically, the effective population size (*N*e) of ancestral populations were twice that of present populations in the refugia, *N*e of the SWEST lineage is the largest, consistent with the diversity value in Table 1, and the estimated times of population divergence match well with the PSMC and previous analyses. The Supplementary Tables 4.2 and 4.3 were revised accordingly.

2) Second, we added new analyses based on whole-genome chloroplast sequence data, as suggested by this reviewer (see the next point below).

3) Third, we performed species distribution modeling (SDM) using updated information on the occurrence record of ginkgo and climate variables. Specifically, we test the effect of multicollinearity among predictor variables and used the variables with relatively lower Pearson correlation coefficients and higher contributions to the model in the SDM analyses. Such a practice is able to avoid the over-fitting of models (Graham, 2003; Pearson et al., 2006). Accordingly, we substantially revised the sections "Environmental adaptation and natural selection" (and Fig. 4) in the text and "Supplementary Note 5. Species distribution modeling" (and Supplementary Table 5.1) in the Supplementary Information.

4) Finally, we added one paragraph and modified many places to expand our discussion on the population genetic structure and dynamics of ginkgo in combination with previous studies in a broader evolutionary context.

A final remark, which I strongly suggest to be considered: Type of raw data should allow to (1) extract complete and highly quality plastid genome information. This data can be used to complement directly past work of the same authors. And (2) the same should be done for mitochondrial DNA. It should be possible to extract at least the entire "gene space". These data might help to interprete dispersal and migration events, since you two additional genomes can be considered.

Response: This is very good point. Actually, it is very easy to obtain the complete chloroplast and mitochondrial genomes of all resequenced individuals, which had been available when the resequencing work was completed. Therefore, as suggested, we assembled 446 chloroplast genomes and performed new analyses based on the whole-genome chloroplast sequence data, including phylogenetic analyses of plastome dataset, the haplotype network analysis and estimates of genetic diversity of the lineages, etc. Accordingly, we presented two new paragraphs and numerous sentences in different sections of the main text to report the results arising from the plastome analyses. Also, one section (Supplementary Note 3a. Chloroplast genome assembly, SNP calling and quality control), two figures (Supplementary Figs. 3.6 and 3.7) and two tables (Supplementary Tables 4.4 and 4.5) were newly added in the revised Supplementary Information. In addition to the major analyses, we provided additional discussion in terms of seed

dispersal and migration events in Discussion. These analyses strengthened our findings involving population genetic structure and dynamic of ginkgo based on maternally inherited genome.

On the other hand, we did not analyze the mitochondrial genomes as suggested by this reviewer, because of the unique characteristics of plant mitochondrial DNAs. First, as demonstrated previously, plant mitochondrial DNAs exhibit low rates of nucleotide substitution, providing limited information for studying population genetics and phylogeny. Second and more importantly, with highly dynamic structure, plant mtDNAs were prone to extensive intramolecular recombination and repeated evolution of multi-chromosomal genomes, causing heteroplasmy and hindering investigation of variation at the whole genome level (reviewed by Schaal 1998. *Mol Ecol*; Wu Z et al. 2015. *PNAS*). This explains why cpDNA markers have been much more widely used than mtDNA markers in plant studies although animal studies rely heavily on the mtDNAs (Schaal 1998; Petit et al. 2005. *Mol Ecol*). Given these facts, we did not use mtDNAs in this case to avoid unnecessary confusions.

In summary, the authors provided a nice story that might have the potential (with their generated raw data) to have some impact on scientific thinking in the field - however, as it stands respective questions and hypotheses have not been raised up ! It is a largely descriptive study.

Response: We appreciate this concern that was also pointed out by reviewer 2. Therefore, we put our findings for ginkgo into broader contexts by highlighting three points: (1) why living fossils appear essentially unchanged, (2) whether living fossils are evolutionary dead ends, and (3) what roles humans have played in the survival and spread of living fossils. In particular, in combination of various studies on ginkgo and other living fossils, we expanded our discussion based on multiple lines of evidence. We believe all these would improve the quality of this work and help further address various questions involving living fossil species in terms of evolutionary history and conservation management.

I do not see any major technical ot statiscal flaws, but this largely depends on reliability of the reference genome, which I cannot comment on.

Response: Thanks for the concern. The chromosome-level genome using the Hi-C approach was included in a parallel manuscript that is currently under review and published simultaneously as a preprint in *BioRxiv* (Zhang et al. Evolution of an XY sex determination mechanism in *Ginkgo biloba* revealed by genome analysis. *bioRxiv*. doi.org/10.1101/517946). The Hi-C approach generated 12 chromosomes spanning 9.03 Gb (~94% of the whole genome). The chromosome-level assembly was well supported by the inter- and intra-chromosome contact matrices of the Hi-C data, and revealed an anti-diagonal pattern within a chromosome and clear separations between chromosomes.

Moreover, chromosome sequence lengths were highly correlated with the physical length of chromosomes (R^2=0.98), further supporting the high quality of our assembly.

Reviewers' comments:

Reviewer #1 (Remarks to the Author):

The authors has carefully addressed all the questions I had. I would recommend the manuscript for publication.

Reviewer #2 (Remarks to the Author):

I thank the authors for carefully considering my recommendations as well as those of all other reviewers. I am confident that the revised manuscript is now greatly improved and is much better than the original submission.

Reviewer #3 (Remarks to the Author):

The authors addressed any of my points very careful and comprehensively. Of course, I did not expect to have this all put into the main manuscript, and they found a good way to have the required improvements split between main document and appendix. The appendix as it stands also reads quite well and should be consulted by the reader!

Congratulations and I hope that the authors appreciated my comments!

Regards
